# Mechanistic insights into IL-6-mediated NK cell dysfunction in NSCLC via the IRE1α-XBP1s-STAT3-UBE2S axis

**DOI:** 10.1038/s41698-025-01140-z

**Published:** 2025-11-18

**Authors:** Yazhen Wang, Zichan Guo, Anqi Xu, Zhaoyue Fu, Yongli Hou, Kang Tang, Juan Li, Feng Zhao, Lihua Chen

**Affiliations:** 1 Department of Immunology, Fourth Military Medical University, Xi’an, Shaanxi China; 2 The College of Life Sciences, Northwest University, Xi’an, Shaanxi China; 3 Department of Pulmonary and Critical Care Medicine, Xijing Hospital, Fourth Military Medical University, Xi’an, China

**Keywords:** Cancer microenvironment, Tumour immunology

## Abstract

Natural killer (NK) cell immunotherapy exhibits limited efficacy in non-small cell lung cancer (NSCLC) due to the suppressive tumor-associated immune microenvironment. Previous studies have shown that interleukin-6 (IL-6) contributes to NK cell dysfunction and decreases NKp30 expression. However, the underlying mechanisms warrant further investigation. In this study, we identified elevated IL-6 and reduced NKp30 expression correlating with NK cell dysfunction and poor prognosis in NSCLC patients. Tumoral IL-6 inversely regulated NKp30 both clinically and in vitro. Mechanistically, IRE1α-XBP1s signaling activated IL-6 transcription via XBP1s binding to the -1201/-300 promoter region. IL-6 induced STAT3-dependent UBE2S upregulation, promoting ubiquitin-mediated NKp30 degradation in NK cells. This dual regulation establishes an XBP1s/IL-6/STAT3-UBE2S axis driving NKp30 loss and functional impairment. Our findings reveal tumor-intrinsic mechanisms suppressing NK cell activity in NSCLC, proposing XBP1s, IL-6, and UBE2S as actionable targets to enhance NK-based immunotherapies.

## Introduction

Lung cancer remains one of the leading causes of cancer-related deaths worldwide and poses a major threat to human health^1–3^. In recent years, the incidence of lung cancer in China has been increasing annually, with non-small cell lung cancer (NSCLC) accounting for approximately 85% of all lung cancer cases^4^. To address this challenge, in addition to traditional surgery, chemotherapy, and radiotherapy, emerging therapies such as molecular targeted therapy and immunotherapy are gradually showing promise as a means of treating this disease^1,5,6^. Although molecular-targeted therapy has become an integral part of NSCLC management, less than 25% of NSCLC patients benefit from targeted therapy, and drug resistance almost invariably develops during the treatment^6–8^. Thus, there is an urgent need to find new strategies to improve the efficacy of NSCLC immunotherapy.

In this context, immunotherapy has become part of the standard of care for NSCLC patients^9^. For example, immune checkpoint inhibitors (ICIs), targeting CTLA4 or PD-L1 pathways, have achieved dramatically improved survival rates and long-term disease control for patients with advanced NSCLC^10^. However, patients with low levels of immune checkpoint molecule expression are less likely to respond to this treatment. Additionally, CAR-T cells in solid tumors face severe challenges, such as target identification, tumor microenvironment with T cell depletion, and heterogeneity of tumor-associated antigens^11^. As an effector cell type capable of killing virus-infected or tumor cells, natural killer (NK) cells have gained prominence in tumor immunotherapy, with NK cell-based immunotherapies representing an increasingly promising approach to tumor management^12–14^.

A small clinical trial published in 2019 demonstrated that the infusion of autologous NK cells is a safe and feasible approach for the treatment of advanced lung cancer, with 85% of patients achieving stable disease within three months of treatment^15^. In the treatment of patients with advanced NSCLC, repeated infusions of active allogeneic NK cells can facilitate disease control in many patients^16^. In a phase I clinical trial, the adaptive transfer of interleukin (IL)-2-activated NK-92 cells demonstrated efficacy in controlling disease progression in lung cancer patients^17^, suggesting that NK cells hold promise as a key component of a multi-pronged treatment strategy for lung cancer.

Although NK cell immunotherapy plays a crucial role in NSCLC, their accumulation, activation and cytotoxic are severely impaired in the immunosuppressive microenvironment. The function of NK cells largely depends on the expression of various NK receptors on their surface, particularly activating receptors such as natural cytotoxicity receptor (NCR), NKG2D, and CD226^18,19^. These receptors can bind to their cognate ligands on the surface of tumor cells, whereupon they send activating signals to regulate the activation and cytolytic activity of NK cells^20^. Nevertheless, NK cells in cancer patients often exhibit reduced expression of activating receptors, particularly NKp30^21–23^, which may be related to the suppressive tumor microenvironment (TME) reshaped by tumor cells^24^. In addition, the expression level of NKp30 was independently correlated with the prognosis of patients with advanced NSCLC^25^. However, the molecular mechanism by which tumor cells affect the differential expression of NKp30 in lung cancer TME remains to be elucidated.

To escape from immune surveillance, tumor cells downregulate the expression of activating receptors and suppress the cytotoxicity of NK cells by secreting a variety of soluble inhibitory factors^24,26,27^, including IL-6, IL-8, and prostaglandin E2 (PGE2). Previous studies have shown that inhibition of IL-6 increased NK cell-mediated killing of human osimertinib-resistant EGFR-mutant NSCLC tumor cells^28^. IL-6 secreted by tumor cells reduced NKp30 expression in NK cells by activating the STAT3 pathway and impaired NK cell function, thereby contributing to the malignancy of oesophageal squamous cell carcinomas^29^. However, the specific mechanism, through which IL-6 inhibits NKp30 expression via the STAT3 pathway remains to be elucidated.

Within the TME, tumor cells can adapt to the local microenvironmental conditions (characterized by hypoxia) by modulating their growth through the unfolded protein response (UPR) triggered by stress from the endoplasmic reticulum (ER)^30^. As a key component of the UPR, inositol-requiring enzyme 1α (IRE1α) homodimerizes and autophosphorylates to activate its RNase domain, which excises a 26-nucleotide intron from X-box binding protein 1 (XBP1) mRNA, generating spliced XBP1 (XBP1s) encoding the transcriptionally active isoform. The IRE1α/XBP1s pathway has been demonstrated to upregulate IL-6 expression in a variety of cellular models. For example, hyperactivation of IRE1α in triple-negative breast cancer cells promotes the production of pro-inflammatory and immunomodulatory cytokines such as IL-6, IL-8, CXCL1, and GM-CSF, thereby affecting the phenotypes and functions of NK cells^31^. Similarly, IRE1α-XBP1s signaling in glioblastoma cells promotes the expression of IL-6, CCL2, and CXCL2^32^. Therefore, we speculate that NSCLC cells in a hypoxic microenvironment may promote IL-6 expression by activating the IRE1-XBP1s pathway. However, whether XBP1s functions as a transcription factor that directly binds to the IL-6 promoter to promote its transcription remains to be confirmed.

In the current study, we found that NKp30 was lowly expressed whereas IL-6 was highly expressed in NSCLC patients, contributing to their poor prognosis. We further found that tumor cells reshaped the TME by activating the IRE1α-XBP1s pathway, which in turn promoted IL-6 expression via XBP1s binding to the IL-6 promoter in the −1201 ~ −300 region. Importantly, we demonstrate for the first time that IL-6 promotes ubiquitin-mediated degradation of NKp30 in NK cells in a STAT3-UBE2S-dependent manner, thereby reducing its surface expression and impairing NK cell function. Our results lay a theoretical foundation for further efforts to restore NK cell function with the goal of improving the prognosis of NSCLC patients.

## Results

### NKp30 expression is downregulated on the surface of NK cells in NSCLC patients

**Fig. 1:**
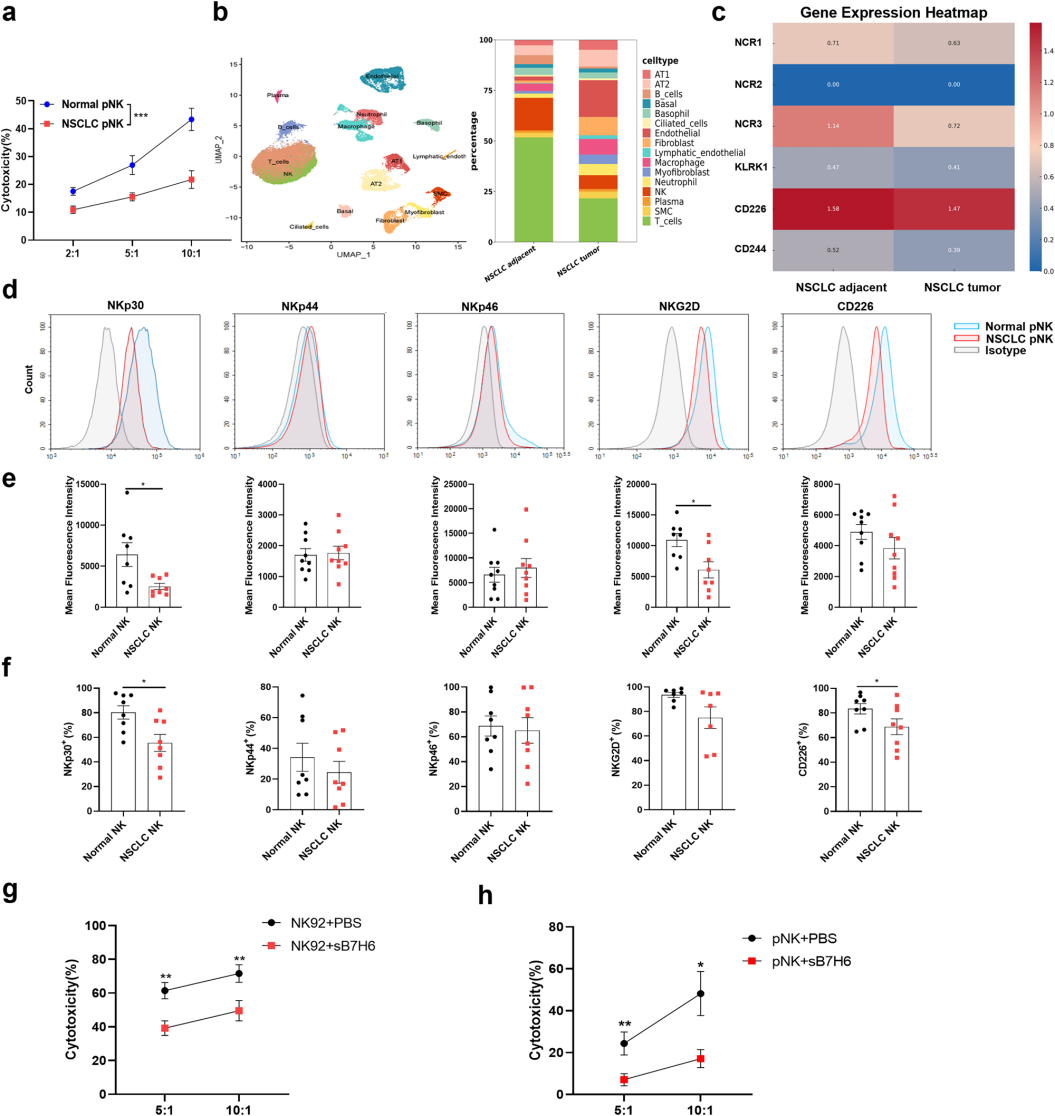
NKp30 expression in NK cells is decreased in NSCLC. **a** pNK cells from healthy volunteers and NSCLC patients were purified for an in vitro cytolytic assay against A549 cells at the indicated effector: target ratio. **b** UMAP plots corresponding to 15 cell types. The proportions of 15 types of cells in cancer and paracancerous tissue samples. **c** Heatmaps displaying the relative enrichment of activating receptors in TiNK cells. **d** Representative histograms of NK cell activating receptor expression levels in pNK cells from healthy volunteers and NSCLC patients. **e** Mean fluorescence intensity values corresponding to NK cell activating receptor expression in pNK cells from healthy volunteers and NSCLC patients. **f** Proportions of NK cell activating receptor-positive NK cells as a fraction of total pNK cells from healthy volunteers and NSCLC patients (n = 8). **g** LDH release assay was used to evaluate the effect of NKp30 blockade with soluble B7H6 for 24 h on NK92 cell function (n = 3). **h** LDH release assay was used to evaluate the effect of NKp30 blockade with soluble B7H6 for 24 h on human peripheral blood-derived NK cell function (n = 4). pNK: peripheral blood NK; NSCLC: non-small cell lung cancer. Data were analyzed using Student’s t tests and one-way ANOVAs. *p < 0.05, ***p < 0.001.

NK cells in solid tumors are phenotypically altered and functionally impaired with a particularly pronounced decrease in the level of activating receptors. To further probe the effect of lung cancer on the phenotype and function of NK cells, the cytotoxicity of peripheral blood NK (pNK) cells from NSCLC patients and healthy volunteers was next compared. The purity of the CD3^-^CD56^+^ NK cells was confirmed by flow cytometry, with an average purity of 96.39% (Supplementary Fig. 1a). Subsequently, the fractions of CD56^dim^CD16^+^ and CD56^bright^CD16^-^NK cells in the total purified NK cell population (Supplementary Fig. 1b) were analyzed, confirming that the proportions of these cells met the experimental requirements. Lactate dehydrogenase (LDH) release assays revealed that the cytotoxic activity of pNK cells against the NSCLC target cell line A549 was suppressed compared to that of pNK cells from healthy volunteers (Fig. 1a), and the expression of the NK cell function-related gene PRF1 (perforin) was significantly reduced in lung adenocarcinoma (LUAD) and lung squamous cell carcinoma (LUSC) (Supplementary Fig. 1c). To explore the altered phenotypic and functional characteristics of NK cells in solid tumors, single-cell RNA sequencing (scRNA-Seq) was performed on tumor and adjacent normal lung tissue samples from three LUAD patients. Unbiased clustering of the cells identified 16 clusters using uniform manifold approximation and projection (UMAP) analysis. Each cluster was annotated using the top-level principal components, and the marker genes associated with each cluster were identified (Fig. 1b). The proportion of clusters representing NK cells was significantly reduced in solid tumors (Fig. 1b). Additionally, the activating receptor-associated genes in the tumor-infiltrating NK (TiNK) cells were broadly downregulated, particularly the activating receptor NKp30 (NCR3) (Fig. 1c). Consistently, flow cytometry results also confirmed that pNK cells from NSCLC patients exhibited a significant reduction in NKp30 expression (Fig. 1d-f). To investigate the involvement of NKp30 in the recognition and cytolysis of lung cancer cells by NK cells, we utilized the soluble ligand B7H6 to preemptively inhibit NKp30 function, followed by assessment of the cytotoxic activity of NK92 cells against a NSCLC cell line. The findings demonstrated a significant reduction in the cytolytic capacity of NK92 cells toward the NSCLC A549 cell line subsequent to NKp30 blockade with soluble B7H6 (Fig. 1g). Similarly, primary human peripheral blood-derived NK cells exhibited markedly diminished cytotoxicity against A549 cells following NKp30 inhibition (Fig. 1h). The results indicate that the activating receptor NKp30 is critically involved in mediating the recognition and lysis of lung cancer cells by NK cells. These results, along with reports that the expression level of NKp30 is independently correlated with the prognosis of patients with advanced NSCLC^25^, suggest that low NKp30 expression on NK cells in NSCLC patients may be associated with NK cell dysfunction and poor prognosis.

### IL-6 is aberrantly secreted at high levels in NSCLC patients

**Fig. 2:**
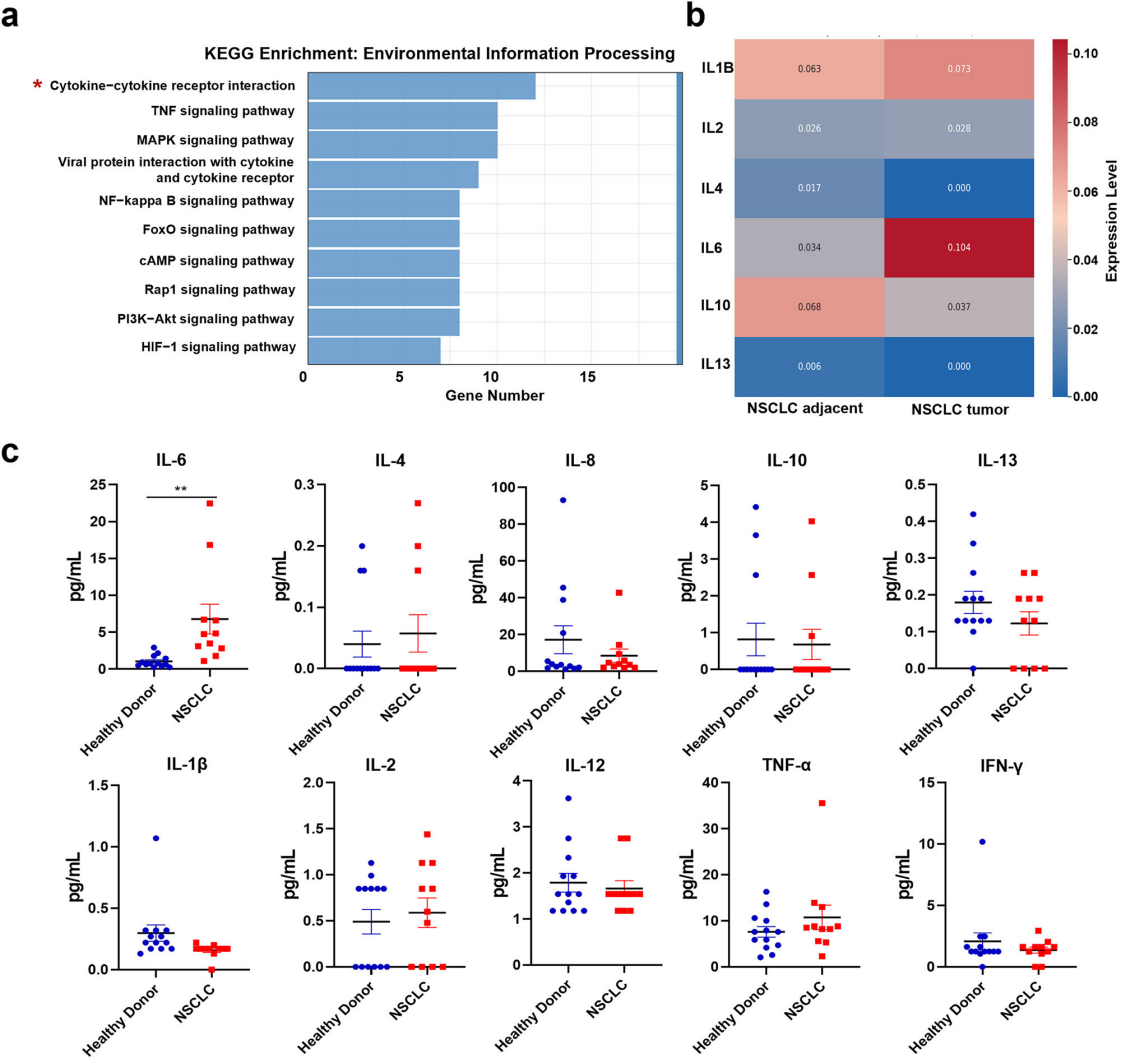
IL-6 level in NSCLC patients is elevated. **a** KEGG enrichment analyses of upregulated genes in NK cells from tumor and paracancerous tissues. **b** The expression levels of related cytokines in NSCLC tumor tissues and paracancerous tissues. **c** Cytokine levels in peripheral blood plasma samples from NSCLC patients (n = 11) and healthy volunteers (n = 13) were measured via Luminex technology. Data were analyzed using Student’s t tests. **p < 0.01.

To investigate the underlying causes of the altered phenotypic function of NK cells in NSCLC patients in vivo, we analyzed differentially expressed genes in NK cells from lung cancer tissues and paracancerous tissues using KEGG pathway enrichment. The results showed that the upregulated differentially expressed genes in TiNK cells from NSCLC patients were enriched primarily in the cytokine-cytokine receptor interaction pathway associated with the processing of environmental information (Fig. 2a).

Given these results, we next examined the transcription levels of tumor cell-related cytokines in NSCLC patients. The results showed that the transcription levels of IL-6 in lung cancer cells were significantly increased (Fig. 2b). We also used Luminex technology to analyze the relative expression levels of cytokines in the peripheral blood plasma of 11 NSCLC patients and 13 healthy volunteers. These findings revealed that only the IL-6 level in the peripheral blood plasma of NSCLC patients was significantly elevated compared to samples from healthy volunteers (Fig. 2c), whereas no differences were observed in the levels of other cytokines with the potential to impact NK cell function, such as IL-4, IL-8, IL-10, or IL-13. No changes were observed in the levels of cytokines that promote NK cell function in NSCLC patients, including IFN-γ, TNF-α, IL-1β, IL-2, and IL-12 (Fig. 2c). These findings suggest that elevated IL-6 levels in NSCLC patients may be associated with their altered NK cell phenotype and function.

### NKp30 expression level shows an inverse correlation with IL-6 in NSCLC patients

**Fig. 3:**
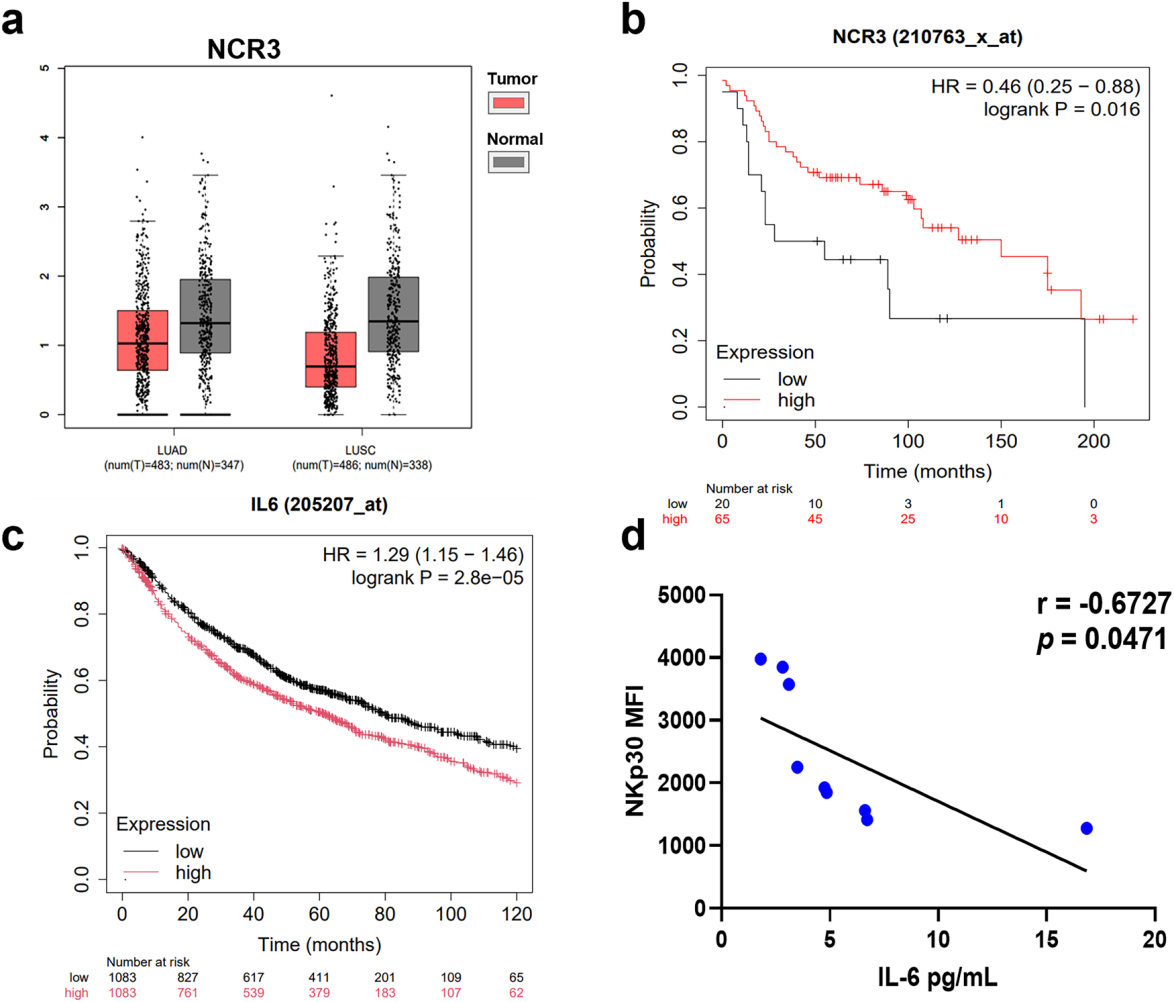
IL-6 levels in NSCLC patients are negatively correlated with NKp30 expression. **a** NCR3 expression in NSCLC tissues from patients stratified by pathological characteristics. **b** Kaplan-Meier survival analyses of NSCLC patients based on the level of NCR3 mRNA expression (NKp30 low, black; NKp30 high, red). **c** Kaplan-Meier survival analyses of NSCLC patients based on the level of IL6 mRNA expression (IL6 low, black; IL6 high, red). **d** The correlation between NKp30 expression and plasma levels of IL-6 in NSCLC patients is shown (n = 9). LUAD: lung adenocarcinoma; LUSC: lung squamous cell carcinoma. Data were analyzed for significance using the log-rank test.

We further used the public data platform GEPIA, based on the TCGA and GTEx databases, to assess the expression level of NKp30 (NCR3) in NSCLC patients. Consistently, NCR3 in LUAD and LUSC showed reduced level compared to normal volunteers (Fig. 3a). In LUAD, NKp30 expression level gradually decreased with tumor progression, whereas in LUSC, that level remained unchanged at all stages (Supplementary Fig. 1d).

Compared to NSCLC patients with high NKp30 expression level, those with low NKp30 expression level exhibited shorter median survival (Fig. 3b). Kaplan-Meier survival analysis showed that patients with high IL-6 level in the body had a shorter median survival than NSCLC patients with lower IL-6 level on the clinically critical 0–120-month window (Fig. 3c), suggesting that IL-6 is a short-to-medium term prognostic biomarker for NSCLC patients. Pearson correlation analysis showed a negative correlation between the expression level of NKp30 on pNK cells and the plasma levels of IL-6 in 9 NSCLC patients (Fig. 3d). These findings indicate that the presence of low levels of NKp30 and high levels of IL-6 in NSCLC patients is associated with a poor prognosis. Furthermore, NKp30 expression levels show a negative correlation with IL-6 expression levels, reinforcing their combined association with poor prognosis in NSCLC patients.

### Tumor cell-derived IL-6 shapes NK cell phenotype with low NKp30 expression

**Fig. 4:**
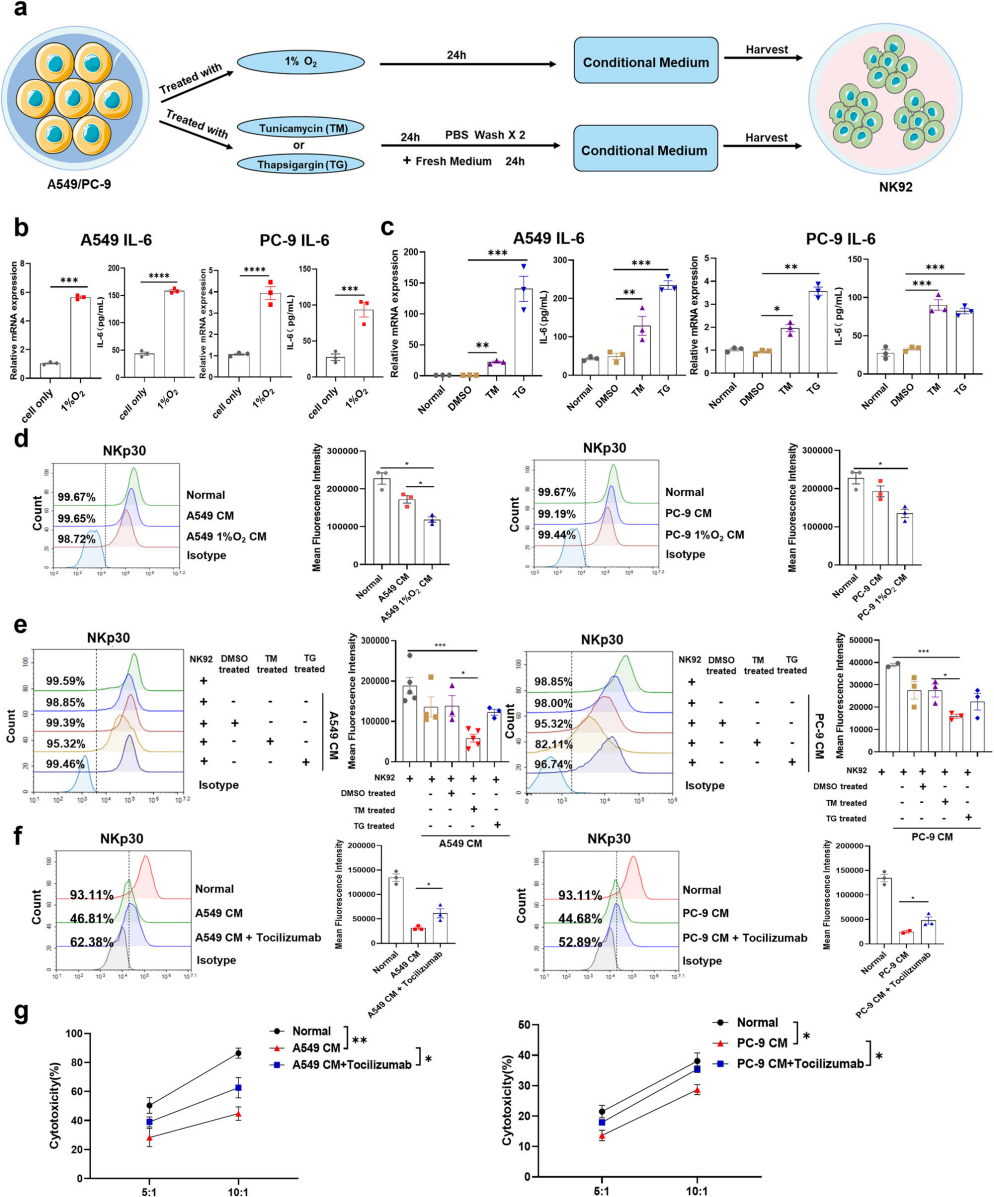
IL-6-derived from tumor cells educates NK cell phenotype with low NKp30 expression. **a** A schematic diagram of NK-92 cells treated with hypoxic CM, A549 TM CM or TG CM. **b** IL6 expression level in A549 and PC-9 cells cultured under hypoxic (1% O_2_) or normoxic conditions for 24 h. Supernatants were collected and IL-6 levels were measured by ELISA (n = 3). **c** IL6 expression level in A549 and PC-9 cells treated using TM or TG for 24 h. Supernatants were collected and IL-6 levels were measured by ELISA (n = 3). **d** Representative histograms and mean fluorescence intensity values corresponding to NKp30 expression in NK-92 cells treated with CM derived from A549 or PC-9 cells cultured under hypoxic (1% O_2_) or normoxic conditions (n = 3). **e** Representative histograms and mean fluorescence intensity values corresponding to NKp30 expression in NK-92 cells treated with A549/PC-9 TM CM or TG CM. **f** Representative histograms and mean fluorescence intensity values corresponding to NKp30 expression in NK-92 cells treated with A549 CM or PC-9 CM with or without tocilizumab. **g** LDH release assay was used to evaluate the effect of conditional medium on NK cell function before and after the use of tocilizumab. TG: thapsigargin; TM: tunicamycin; CM: conditioned medium. Data were analyzed using Student’s t tests and one-way ANOVAs. *p < 0.05, **p < 0.01, ***p < 0.001, ****p < 0.0001.

Given the high level of IL-6 in NSCLC patients, we cultured NSCLC cell lines under hypoxic conditions (1% O_2_) to better simulate the tumor microenvironment (TME) in vivo in solid tumors. Stressors such as hypoxia, nutrient deficiencies, and acidosis in the tumor microenvironment can lead to ER stress in tumor cells, which in turn remodel the microenvironment by activating the UPR response^33,34^. Previous studies have shown that UPR is involved in regulating the tumor immunological microenvironment and inhibiting the functionality of anti-tumor immune cells in patients with multiple solid tumors^33,35,36^. To better mimic the real tumor microenvironment, we also treated NSCLC cell lines with the classical ER stress inducers thapsigargin (TG) or tunicamycin (TM) for 24 h, washed them twice with PBS, and then added fresh medium for an additional 24 h. The supernatant in above three models was then collected as conditioned medium (CM) to culture NK92 cells for 24 h (Fig. 4a).

We found that lung cancer cell lines secreted a large amount of IL-6 under hypoxic conditions (Fig. 4b). Moreover, both qPCR and Western blotting demonstrated that the levels of BIP and IRE1α were significantly increased in A549 and PC-9 cells after 48 hours of treatment (Supplementary Fig. 2a, b), confirming successful UPR induction and activation of the IRE1α-XBP1s pathway. Consistent with these findings, we found that UPR status NSCLC cells also secreted IL-6 in large quantities, similar to the in vivo status of NSCLC patients (Fig. 4c). Flow cytometry results showed that CM from hypoxia-exposed lung cancer cells more significantly reduced the expression of NKp30 on the surface of NK-92 compared to normal culture (Fig. 4d, Supplementary Fig. 2c, d). Flow cytometry results also showed that CM derived from A549 cells in UPR status more significantly inhibited NKp30 expression compared to CM derived from these NSCLC cells without TM/TG treatment, with similar findings observed for CM prepared from PC-9 cells (Fig. 4e, Supplementary Fig. 2e, f).

To further evaluate the inhibitory effect of IL-6 on NKp30 expression in NK cells within the TME, we used Tocilizumab, an IL-6R neutralizing antibody, to block the effect of IL-6 on NK cells. Flow cytometry results showed that the reduced NKp30 expression on the surface of NK cells was significantly restored after Tocilizumab treatment, compared to the A549 CM group (Fig. 4f). After the addition of PC-9 CM and Tocilizumab, NKp30 expression was significantly increased compared to that observed following treatment with PC-9 CM alone (Fig. 4f). Moreover, the LDH release assay showed a partial reversal of the inhibition of NK cell killing activity by CM following Tocilizumab treatment (Fig. 4g). To further elucidate the role of IL-6 on NK cells within the NSCLC microenvironment, NK92 cells were treated with recombinant IL-6 protein, followed by an assessment of the expression levels of activating receptors and their cytotoxic function. The findings demonstrated that IL-6 markedly decreased the expression of the activating receptor NKp30 on NK92 cells, while the expression of the other three activating receptors remained unaffected (Supplementary Fig. 3a–c). Additionally, IL-6 significantly suppressed the cytotoxic activity of NK92 cells against NSCLC cell lines A549 and PC-9 (Supplementary Fig. 3d, e). Notably, silencing IL-6 expression in PC-9 cells via siRNA (Supplementary Fig. 3f, g) substantially reversed the inhibitory effect of the CM on NKp30 expression (Supplementary Fig. 3h-j).

Next, human peripheral blood NK cells were employed to further validate the impact of IL-6 within the tumor microenvironment on the expression of NKp30 and the cytotoxic function of these cells. The results demonstrated that IL-6 markedly suppressed the expression of NKp30 on the surface of human peripheral blood NK cells, while the expression levels of other activating receptors remained unaffected (Supplementary Fig. 4a-c). Similarly, conditioned medium derived from two NSCLC cell lines, A549 and PC-9, significantly decreased NKp30 expression on these NK cells without altering the expression of other activating receptors (Supplementary Fig. 4d–f). Notably, both recombinant IL-6 and tumor-conditioned medium substantially diminished the cytotoxic activity of human peripheral blood NK cells against A549 cells (Supplementary Fig. 4g, h).

These findings suggest that the elevated level of IL-6 within the NSCLC tumor microenvironment suppresses NKp30 expression on the surface of NK cells, thereby impairing their cytotoxicity.

### Tumor cells increase IL-6 expression by activating IRE1α-XBP1s pathway

**Fig. 5:**
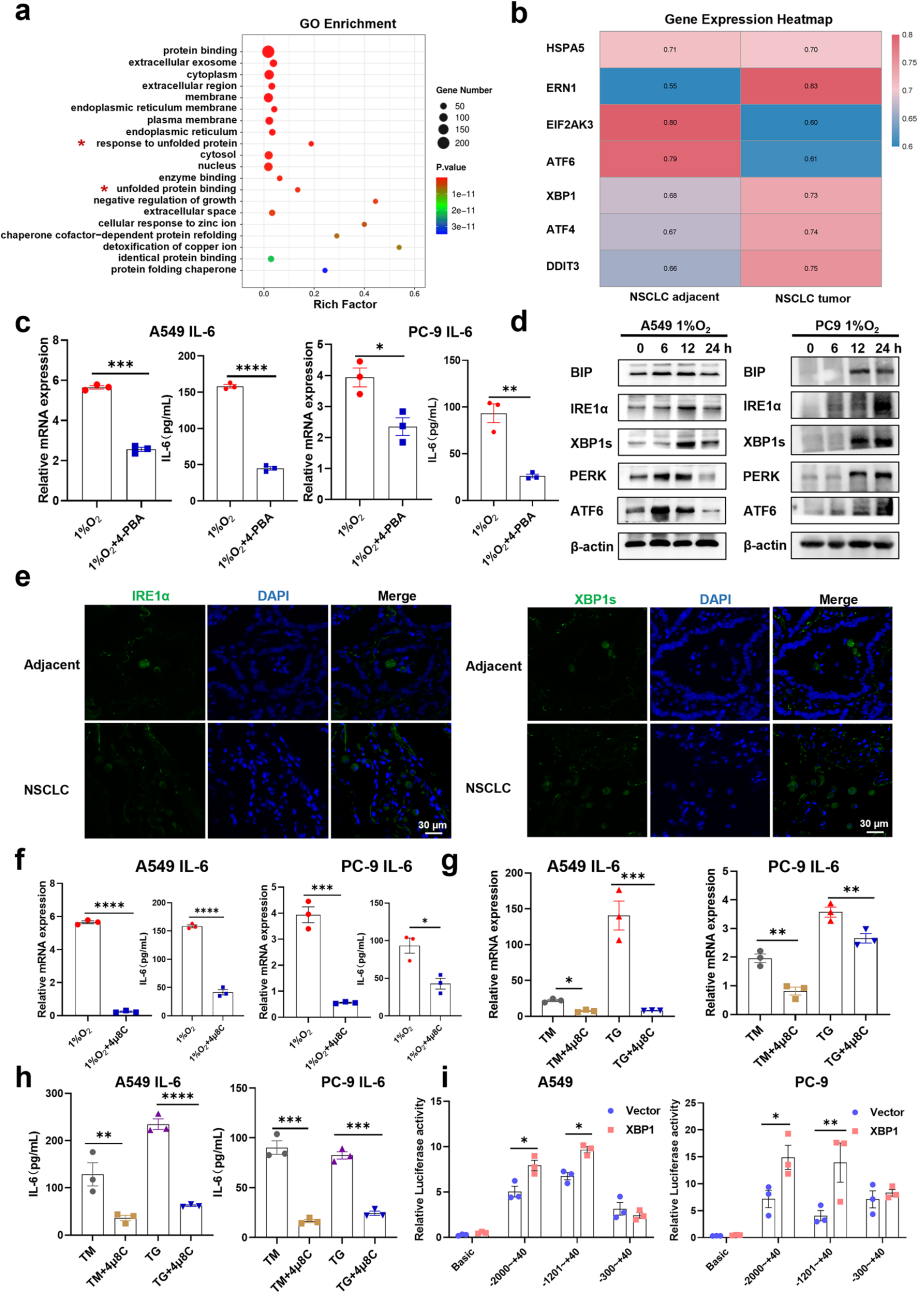
Tumour cells massively secrete IL-6 through activation of the IRE1α-XBP1s axis. **a** GO enrichment analysis was used to analyse the top 20 pathways of up-regulated differential genes in NSCLC tumour tissues. **b** Heatmaps displaying the relative enrichment of UPR marker genes in NSCLC tumour tissues. **c** IL6 expression level in A549 and PC-9 cells under 1% O_2_ conditions with or without 4μ8C for 24 h. Supernatants were collected and IL-6 levels were measured by ELISA (n = 3). **d** UPR marker genes expression level in A549 and PC-9 cells under 1% O_2_ conditions for the indicated times. **e** Representative IRE1α and XBP1s staining in NSCLC tissues. Scale bar, 30 μm. **f** IL6 expression level in A549 and PC-9 cells under 1% O_2_ conditions with or without 4μ8C for 24 h. Supernatants were collected and IL-6 levels were measured by ELISA (n = 3). **g**, **h** IL6 expression level in A549 and PC-9 cells treated using TM/TG with or without 4μ8C for 24 h and then cultured with normal medium for 24 h. Supernatants were collected and IL-6 levels were measured by ELISA (n = 3). **i** Luciferase activity of A549 cells and PC-9 cells transfected with the indicated reporters and XBP1-expressing or control vectors (n = 3). UPR: unfolded protein response; TG: thapsigargin; TM: tunicamycin. Data were analyzed using one-way ANOVAs. *p < 0.05, **p < 0.01, ***p < 0.001, ****p < 0.0001.

Given the critical role of IL-6 in suppressing NKp30 expression and impairing NK cell cytotoxicity, we investigated the mechanisms underlying IL-6 upregulation in NSCLC. GO enrichment analysis revealed that the up-regulated differential genes in tumor tissues were enriched primarily in UPR-related pathways, such as unfolded protein binding, protein folding, and the response to unfolded protein (Fig. 5a). Consistently, UPR-related marker genes were generally upregulated in NSCLC cells, including those encoding IRE1 (ERN1), ATF4 (ATF4), XBP1 (XBP1), and CHOP (DDIT3) (Fig. 5b). To ascertain whether tumor cells promote the secretion of IL-6 by activating UPR, we employed the ER stress inhibitor 4-PBA to inhibit the UPR. This inhibition reduced the ability of NSCLC cells to secrete IL-6 under hypoxic conditions (Fig. 5c).

To further determine the specific pathway of UPR activation in tumor cells of NSCLC patients, we examined the activation of the three UPR pathways in NSCLC cells at different time points under hypoxic conditions in a simulated tumor microenvironment in vitro. The results showed that the IRE1α-XBP1s pathway in NSCLC cells was significantly activated following long-term hypoxia treatment (Fig. 5d, Supplementary Fig. 5a). Immunofluorescence results showed that the expression levels of IRE1α and XBP1s in NSCLC tumor tissues were also significantly increased compared to that of the other two key genes, ATF6 and PERK, in the UPR pathway (Fig. 5e, Supplementary Fig. 5b, c). Additionally, the ability of tumor cells to secrete substantial quantities of IL-6, triggered by hypoxia and ER stress inducer, was suppressed in an in vitro model following the utilization of the IRE1α-XBP1s pathway inhibitor, 4μ8C (Fig. 5f-h). Furthermore, an XBP1-binding site in the IL-6 promoter was identified, and luciferase reporter assays showed that the binding site located at -1201 to -300 was responsible for XBP1s-mediated transcriptional activity (Fig. 5i). To investigate whether HIF1α, a principal transcription factor activated under hypoxic conditions within the tumor microenvironment, contributes to the regulation of IL-6 production, we employed siRNA-mediated knockdown of HIF1α in PC-9 cells followed by exposure to hypoxia. Subsequent analysis of IL-6 mRNA expression and protein secretion revealed that suppression of HIF1α did not significantly alter IL-6 transcription or secretion levels in PC-9 cells (Supplementary Fig. 5d-f). These findings suggest that NSCLC cells within the tumor microenvironment produce elevated levels of IL-6 through mechanisms independent of HIF1α activity. Overall, these findings confirm that NSCLC cells secrete large amounts of IL-6 by activating the IRE1α-XBP1s pathway, which reduces NKp30 surface expression on NK cells and impairs their function.

### IL-6 promotes ubiquitin-mediated degradation of NKp30

**Fig. 6:**
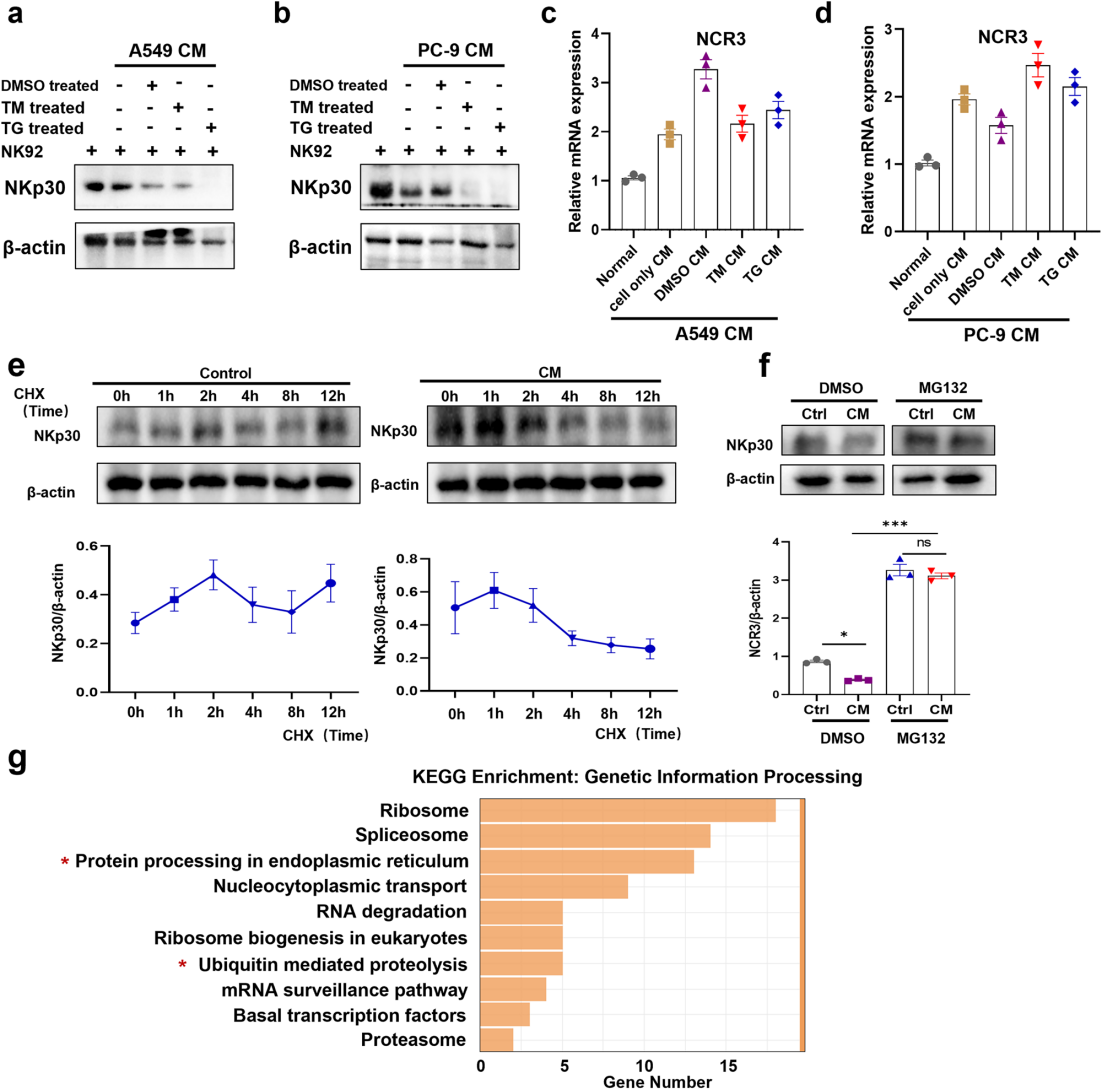
IL-6 promotes ubiquitination and degradation of NKp30. **a**–**d** NCR3 expression levels in NK-92 cells treated with A549 (**a**, **c**)/PC-9 (**b**, **d**) TM CM or TG CM (n = 3). **e** NK-92 cells were treated with normal medium or CM and stimulated with CHX (100 ng/ml) for the indicated amount of time, after which NKp30 levels were detected via Western blotting (n = 3). **f** NK-92 cells were treated with DMSO or MG132 (20 mM) for 12 h, after which NKp30 was detected by Western blotting. **g** KEGG enrichment analyses of upregulated genes in NK cells from tumor and paracancerous tissues in NSCLC. CHX: cycloheximide; DMSO: dimethyl sulfoxide; TG: thapsigargin; TM: tunicamycin; CM: conditioned medium. Data were analyzed using one-way ANOVAs. *p < 0.05, ***p < 0.001.

To further explore the mechanism by which IL-6 inhibits NKp30 expression, we first examined the protein and mRNA levels of NKp30 (NCR3) in CM-treated NK cells. Surprisingly, however, the protein and mRNA expression levels of NKp30 in NK cells showed inconsistent trends after treatment with different CMs. In comparison to NK92 cells cultured in CM derived from conventionally maintained A549 cells, NK92 cells exposed to CM from A549 cells treated with TM/TG exhibited a significant reduction in NKp30 protein expression (Fig. 6a). Notably, this decrease in protein levels was not accompanied by a corresponding change in NKp30 mRNA expression (Fig. 6c). The alterations in NKp30 transcription and expression levels observed in NK92 cells exposed to CM derived from PC-9 cells under varying conditions are consistent with the changes detected in NK92 cells treated with CM from A549 cells subjected to similar conditions (Fig. 6b, d), suggesting that NKp30 expression was not regulated at the transcriptional level.

To investigate this further, we speculated that the reduction in NKp30 protein levels under these conditions might be related to post-transcriptional regulatory mechanisms, such as ubiquitin-mediated proteasomal degradation. To validate this speculation, we sequentially examined changes in NKp30 protein expression in NK-92 cells following treatment with the protein synthesis inhibitor cycloheximide (CHX) and the proteasome inhibitor MG-132. The results revealed that NKp30 degradation in NK92 cells was accelerated by A549 CM following CHX treatment (Fig. 6e), whereas this degradation was inhibited by MG-132 (Fig. 6f), suggesting that IL-6 in the CM may reduce NKp30 expression by promoting its accelerated degradation.

In addition, the up-regulated genes in TiNK cells were primarily enriched in protein processing in the endoplasmic reticulum and the ubiquitin-mediated proteolysis pathways associated with genetic information processing (Fig. 6g). Collectively, these data indicate that the ubiquitin-mediated proteolysis pathway may be involved in IL-6-mediated downregulation of NKp30 in NSCLC patients.

### IL-6 promotes NKp30 ubiquitination and degradation via the STAT3-UBE2S axis

**Fig. 7:**
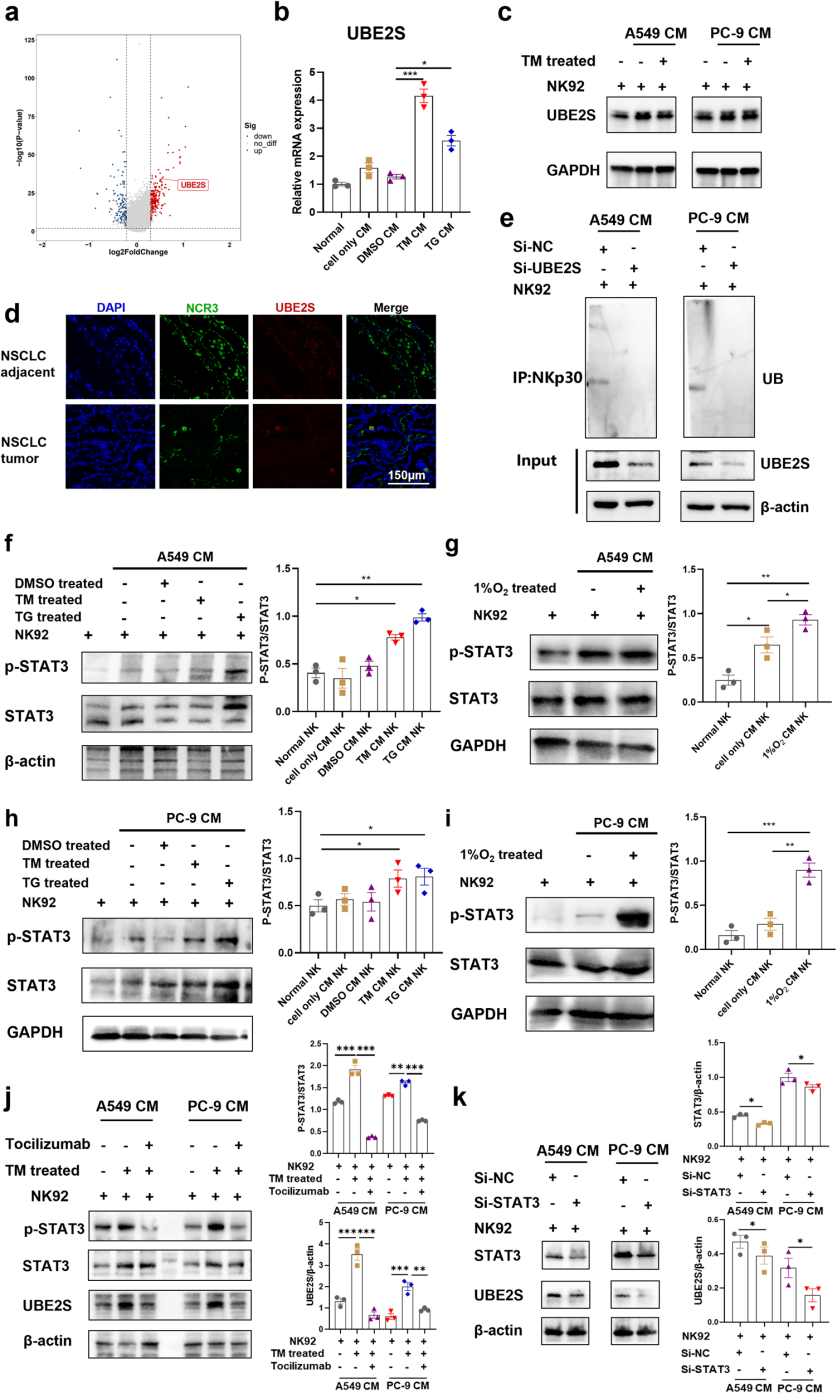
IL-6 promotes ubiquitinated degradation of NKp30 in NK cells via the STAT3-UBE2S axis. **a** Volcano plots showing differentially expressed genes when comparing tumors and normal paracancerous tissues in NSCLC. Genes exhibiting an adjusted *p*-value < 0.05 were considered significant. **b**, **c** UBE2S expression levels in NK92 cells treated with A549 TM CM or TG CM (n = 3). **d** Representative NCR3 and UBE2S staining in NSCLC tissues. Scale bar, 150 μm. **e** UBE2S-deficiency impairs IL-6-induced ubiquitination of NKp30. Control or UBE2S-deficient NK92 cells were stimulated with A549 CM or PC-9 CM for 24 h before co-immunoprecipitation and immunoblotting analysis with the indicated antibodies. **f** Representative Western blots showing STAT3 and p-STAT3 levels in NK-92 cells treated with A549 TM CM or TG CM. **g** Representative Western blots showing STAT3 and p-STAT3 levels in NK-92 cells treated with A549 1%O_2_ CM. **h** Representative Western blots showing STAT3 and p-STAT3 levels in NK-92 cells treated with PC-9 TM CM or TG CM. **i** Representative Western blots showing STAT3 and p-STAT3 levels in NK-92 cells treated with PC-9 1%O_2_ CM. **j** The phosphorylation level of STAT3 protein and the expression level of UBE2S were analyzed by Western blot after NK92 cells were treated for 24 h according to the labeling conditions. Tocilizumab, 10 μg/mL. **k** STAT3-deficiency down-regulates the level of UBE2S. Control or STAT3-deficient NK92 cells were treated with A549 CM or PC-9 CM for 24 h before immunoblotting analysis with the indicated antibodies. TG thapsigargin, TM tunicamycin, CM conditioned medium. Data were analyzed using one-way ANOVAs. *p < 0.05, **p < 0.01, ***p < 0.001.

Preliminary single-cell RNA sequencing (scRNA-Seq) analysis of tumor-infiltrating natural killer (TiNK) cells derived from NSCLC patients indicated a significant upregulation of the ubiquitin-conjugating enzyme E2S (UBE2S) (Fig. 7a). In light of this finding, the transcriptional expression of UBE2S was examined in NK-92 cells cultured under different conditions, revealing a marked increase in UBE2S mRNA levels in cells maintained in conditioned medium (CM) (Fig. 7b). Correspondingly, protein expression analysis demonstrated a significant elevation of UBE2S in NK cells cultured in CM (Fig. 7c). Immunofluorescence staining further corroborated the enhanced expression of UBE2S in TiNK cells (Fig. 7d). Additionally, analysis of publicly available datasets revealed a significant upregulation of UBE2S in lung adenocarcinoma (LUAD) and lung squamous cell carcinoma (LUSC) tissues (Supplementary Fig. 6a). Survival analysis indicated that NSCLC patients exhibiting high UBE2S expression had a significantly reduced median survival compared to those with low expression levels (Supplementary Fig. 6b). Moreover, immunoprecipitation assays demonstrated that NKp30 underwent substantial ubiquitination in NK cells following CM treatment, an effect that was markedly attenuated upon UBE2S knockdown (Fig. 7e).

The activation of the STAT3 signaling pathway subsequent to IL-6 stimulation was examined via western blot analysis. The findings demonstrated a significant increase in STAT3 phosphorylation in NK92 cells after 2 h of incubation with CM derived from A549 TM or A549 TG cells, relative to the control group (Fig. 7f, Supplementary Fig. 6c). Similarly, STAT3 phosphorylation levels in NK92 cells were markedly elevated following exposure to CM obtained from A549 cells cultured under hypoxic conditions (Fig. 7g). Furthermore, alterations in phosphorylated STAT3 (p-STAT3) levels in NK92 cells treated with various CM preparations derived from PC-9 cells were largely consistent with those observed in the A549 CM-treated group (Fig. 7h, i).

To investigate whether IL-6 facilitates the ubiquitin-mediated degradation of NKp30 via the STAT3-UBE2S signaling pathway, IL-6 receptor (IL-6R) was inhibited using Tocilizumab in NK cells cultured in CM. This intervention resulted in a significant reversal of both STAT3 phosphorylation and UBE2S upregulation (Fig. 7j). Furthermore, knockdown of STAT3 through siRNA markedly decreased UBE2S expression in CM-treated NK cells (Fig. 7k). Collectively, these results indicate that elevated IL-6 levels within the NSCLC tumor microenvironment promote the ubiquitination and subsequent degradation of NKp30 in NK cells by activating the STAT3-UBE2S signaling axis, thereby impairing NK cell cytotoxic function.

### Targeting STAT3/UBE2S/NKp30 enhances NK cell function and suppresses NSCLC tumor growth

To determine the role of the STAT3/UBE2S/NKp30 axis in vivo, we established a PC-9 subcutaneous tumor model using C-NKG mice deficient in T cells, B cells, and NK cells. Upon tumor establishment reaching ~100 mm³, mice received intravenous administrations of NK92 cells across distinct experimental groups. Following the treatment period, mice were euthanized via carbon dioxide asphyxiation, and subsequent analyses were conducted to assess tumor progression, expression profiles of surface receptors on tumor-infiltrating NK92 cells, and alterations in their cytotoxic functionality. The data demonstrated that administration of the IL-6 receptor-neutralizing antibody Tocilizumab significantly suppressed tumor volume expansion (Supplementary Fig. 7a, b). Notably, this inhibitory effect was abrogated after blocking the function of NKp30 on the surface of NK92 cells with the soluble ligand B7H6. Further examination revealed that Tocilizumab treatment markedly upregulated NKp30 expression on NK92 cells within the tumor microenvironment (Supplementary Fig. 7c) and enhanced their granzyme B secretion capacity (Supplementary Fig. 7d). Conversely, simultaneous blockade of NKp30 with B7H6 negated these therapeutic benefits. Collectively, these findings underscore the potential of targeting the STAT3/UBE2S/NKp30 axis as a viable approach to impede NSCLC tumor growth and improve clinical outcomes.

## Discussion

NK cell immunotherapy plays a crucial role in controlling the progression of solid tumors, particularly NSCLC^37^. Multiple immunosuppressive mechanisms contribute to NK cells dysfunction in solid tumors, including inhibitory cytokines (e.g., IL-6, IL-8, PGE2, etc.) derived from tumor cells or myeloid-derived suppressor cells, high expression of inhibitory receptor ligands, or deficient expression of activating receptor ligands on tumor cells^24,26,27,38^. The cytotoxic function of NK cells against tumor cells is frequently contingent upon the expression of diverse NK cell surface receptors, particularly activating receptors including CD226 (also referred to as DNAM-1), NKG2D, and natural cytotoxicity receptors (NCRs), which encompass NKp30, NKp44, and NKp46. Engagement of these activating receptors with their specific ligands initiates intracellular signaling cascades within NK cells via immunoreceptor tyrosine-based activation motifs (ITAMs) located in their cytoplasmic domains or through associated adaptor molecules containing ITAMs. This signaling facilitates NK cell-mediated tumor cell lysis and the secretion of cytokines^18^. Activating receptors that either possess ITAM motifs or form dimers with ITAM-bearing molecules such as CD3ζ or FcRγ include NKp30, NKp44, NKp46, CD16, and KIR-S^20^. In patients with early-stage NSCLC, tumor-infiltrating NK cells demonstrate diminished expression of activating receptors (NKp30, NKp80, CD16, and CD226) alongside reduced degranulation capacity and cytokine production^39^. Conversely, in advanced NSCLC, transcriptional levels of NCRs are markedly downregulated, with NKp30 expression uniquely correlating independently with patient prognosis^25^. Structurally, NKp30 comprises an extracellular immunoglobulin V-like domain and a transmembrane region capable of associating with CD3ζ or FcRγ chains^40^. This receptor is predominantly expressed on mature NK cells as well as innate lymphoid cell subsets type 2 (ILC2) and type 3 (ILC3). NKp30 recognizes both microbial and tumor-associated ligands, including BAT3 and the B7 family member B7-H6, thereby enhancing the susceptibility of tumor cells to NK cell-mediated cytotoxicity^41^. In this study, the observed reduction in NKp30 expression on NK cells from NSCLC patients aligns with previously reported changes in NK cells within the solid tumor TME^21,22,29^. Interestingly, the elevated IL-6 was observed in both scRNA-Seq and peripheral blood of NSCLC patients, whereas NKp30 expression was negatively correlated with IL-6 expression levels in these patients. Our results align with previous studies showing that NSCLC patients have elevated levels of plasma IL-6^42^. Thus, we postulate that high IL-6 expression is closely associated with low NKp30 expression in NK cells, facilitating NK cell dysfunction and poor prognosis in NSCLC patients in vivo.

Previous studies have reported that culturing a variety of cells under 1% O₂ conditions can promote high IL-6 expression^43–45^. In addition, 4-phenyl butyric acid (4-PBA), a classical ER stress inhibitor that also prevents activation of the NF-κB pathway, reduced the release of the pro-inflammatory mediators IL-6^46^. Moreover, treatment of mouse astrocytes with classical ER stress inducers, thapsigargin (TG) and tunicamycin (TM), can promote the expression of IL-6^47^. To elucidate the in vivo relevance of IL-6-mediated impairment of NKp30 expression in NSCLC, our in vitro simulations—based on established models of hypoxia and ER stress—demonstrate that elevated IL-6 levels directly suppress NKp30 on NK cells. This mechanism is reinforced by multiple lines of evidence: altered transcript profiles under UPR conditions, elevated IL-6 in conditioned medium, and functional rescue using the IL-6R inhibitor Tocilizumab. Importantly, these findings align with scRNA-seq data revealing enrichment of cytokine-cytokine receptor pathways in tumor-infiltrating NK cells, suggesting IL-6 as a key modulator of NK cell dysfunction in the tumor microenvironment. However, the critical role of IL-6 remains to be validated in murine models, highlighting a gap for future investigation.

Building upon the observed inverse correlation between NKp30 expression and IL-6 levels in NSCLC patients in vivo, as well as the in vitro evidence demonstrating IL-6-mediated suppression of NKp30 surface expression, this study elucidates a key mechanism underlying this impairment. Our findings indicate that IL-6 primarily impacts NKp30 through post-transcriptional regulation, specifically by promoting its ubiquitination and degradation. This conclusion is supported by the observed dysregulation of NCR3 mRNA alongside experimental confirmation using proteasomal inhibitors (MG-132) and translational blockade (CHX) in vitro, consistent with patterns seen in patient-derived single-cell sequencing data. Critically, we identified UBE2S, a ubiquitin-conjugating enzyme upregulated in tumor-infiltrating NK (TiNK) cells from NSCLC patients and our in vitro models, as a likely mediator of this process. Given UBE2S’s established roles in tumor progression^48–51^ and its reported crosstalk with the STAT3 pathway^52,53^, and considering the known regulation of NK cell STAT3 by cytokines including IL-6^29,54,55^ and its involvement in cytokine-driven NK receptor downregulation (e.g., in esophageal squamous carcinoma^29^), we hypothesized and subsequently validated that IL-6 impairs NK cell function within the tumor microenvironment by activating the STAT3-UBE2S axis to enhance NKp30 ubiquitination and degradation. This proposed STAT3-UBE2S-NKp30 axis aligns well with our enrichment analysis showing ubiquitin-mediated proteolysis as a key pathway in TiNK cells. While this study establishes a functional link between IL-6, STAT3, UBE2S, and NKp30 degradation, the precise molecular mechanism by which STAT3 regulates UBE2S expression warrants further investigation.

Hypoxia, nutrient deprivation, and acidosis in NSCLC microenvironment are well-established inducers of ER stress^30,45^. Given prior evidence linking ER stress, particularly activation of the IRE1α pathway, to increased IL-6 secretion in other cell types (e.g., glial cells under ischemic conditions)^56–58^, we investigated whether a similar mechanism drives IL-6 production in NSCLC tumor cells. Critically, our study demonstrates that ER stress and the IRE1α-XBP1s axis are key regulators of IL-6 in this context. Inhibition of ER stress (4-PBA) or specifically IRE1α (4μ8C) significantly reduced IL-6 secretion, and findings further corroborated by elevated IRE1α and XBP1s expression in NSCLC tumor tissues from single-cell sequencing data. Most importantly, we identified a direct molecular link: XBP1s, generated via IRE1α activation, binds to a specific region (−1200 ~ −300) of the IL-6 promoter to drive its transcription. Therefore, our data establish the IRE1α-XBP1s pathway as a central mechanism for IL-6 overexpression in NSCLC tumors. This mechanistic insight highlights the potential of targeting this axis, including exploring the repurposing of ER stress modulators, as a rational therapeutic strategy for high-IL-6 NSCLC.

Previous research has shown that while autologous NK cell therapy is a viable approach, it fails to achieve the desired anti-tumor effects. Some scientists suggest that the impaired function of autologous NK cells may result from the binding of self-MHC molecules, expressed on the surface of tumor cells, to inhibitory KIRs^59^. These inhibitory KIRs transmit inhibitory signals and prevent the activation of NK cells. In this context, the NK92 cell line---which lacks the majority of currently known inhibitory KIR receptors and exhibits low expression of KIR2DL4---has brought new hope for NK cell immunotherapy. The NK92 cell line, as an immortalized cell line derived from lymphoma patients, expresses a great variety of NK cell activating receptors, including NKp30, NKp46, NKG2D, and others^60^, and exhibits potent cytotoxicity against a variety of malignant tumor cells^61^. As a continuously growing NK cell line, NK92 can be expanded in vitro in the presence of IL-2, thereby providing a consistent supply of highly cytotoxic NK cells, which is an optimal prerequisite for adoptive NK cell transfer therapy. Previous studies have demonstrated that the infusion of NK92 cell lines for the treatment of various tumor types has proven to be safe and effective^62,63^. Accordingly, the NK92 cell line was used in this study to investigate the effects of endoplasmic reticulum stress on the expression of NK cell-activating receptors and cytotoxic activity in tumor patients. The NK92 cell line provides a theoretical and experimental basis for further research into the mechanisms underlying NK cell phenotypic alterations and dysfunction in tumor patients, as well as for improving the efficacy of adoptive NK92 cell transfer in tumor patients.

**Fig. 8:**
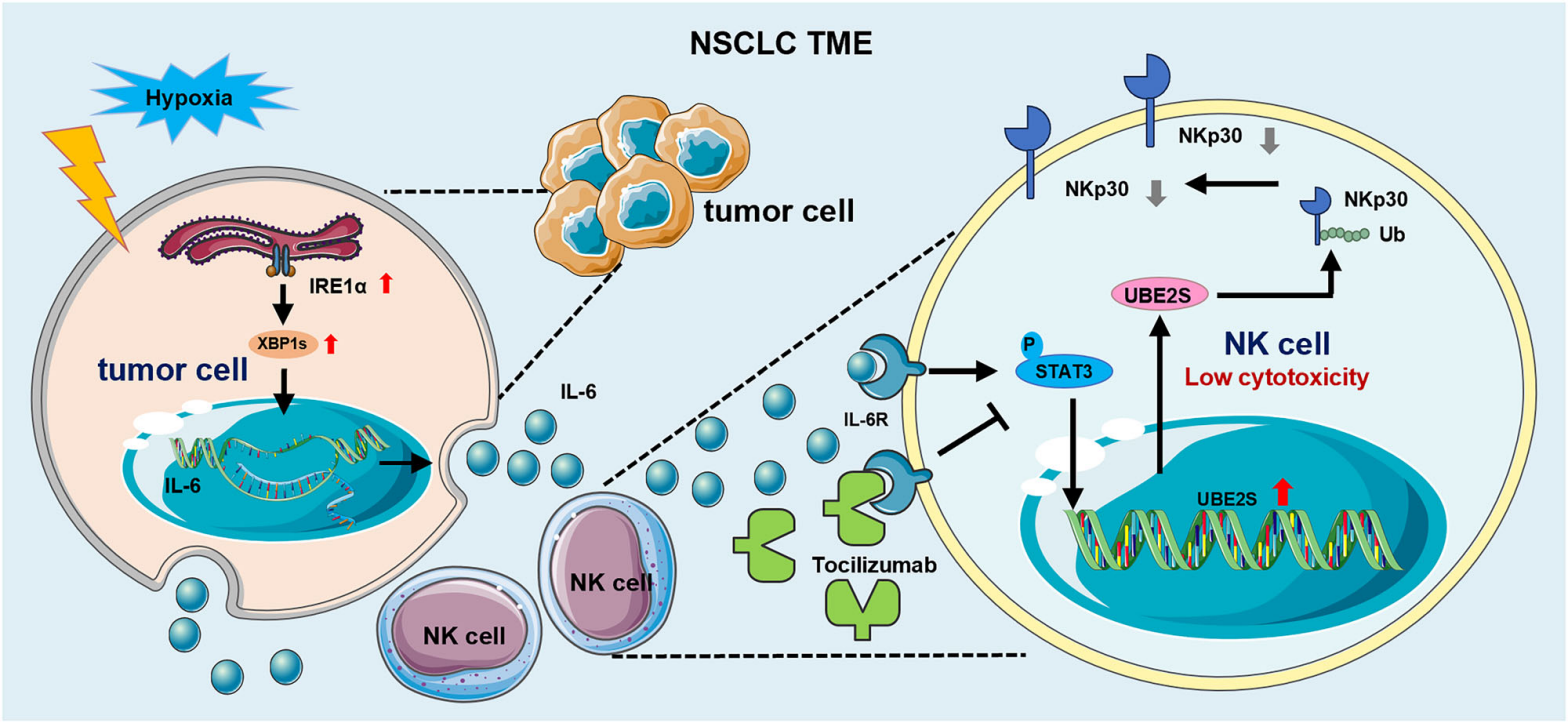
A schematic diagram depicting hypoxia driven IL-6-inducde NK dysfunction in NSCLC. IRE1α, an endoplasmic reticulum stress sensor, was activated under the hypoxia tumor microenvironment, thereby with enhanced IL-6 secretion via the binding between downstream XBP1s and the promoter of IL-6 in tumor cells. Then, the phosphorylation of STAT3 triggered by highly expressed IL-6 stimulated the transcriptional level of UBE2S, thus resulting in NKp30 ubiquitination degradation and NK dysfunction.

In summary, our findings provide preliminary insights into the molecular mechanisms through which ER stress regulates the differential expression of NK cell-activating receptors, thereby impairing the cytotoxicity of NK cells in NSCLC. Specifically, NSCLC cells activate the IRE1α arm of the UPR pathway in response to stressors in the tumor microenvironment, which in turn promotes IL-6 secretion. Consequently, elevated IL-6 levels activate the STAT3 phosphorylation in NK cells, upregulating UBE2S expression and accelerating the ubiquitin-mediated degradation of NKp30 (Fig. 8). Taken together, these findings have identified a novel therapeutic target for improving NK cell-based immunotherapy in NSCLC.

## Methods

### Human samples

Human peripheral blood NK cells were isolated and purified from peripheral blood samples from healthy volunteers provided by the Department of Blood Transfusion, Xijing Hospital, Xi’an, China, while samples from NSCLC patients were collected by the Department of Respiratory Medicine, Xijing Hospital, Xi’an, China. We collected peripheral blood specimens from 12 NSCLC patients in total aged 51–66 years with a male-to-female ratio of 1:1. These patients were pathologically diagnosed with NSCLC prior to inclusion in the study, including 6 cases of LUAD and 6 cases of LUSC. They had not undergone radiotherapy, had no history of chronic diseases such as diabetes or hypertension, and had no restrictions regarding TNM staging. The control group consisted of 12 age-matched healthy volunteers with the same male-to-female ratio. Cancer and paracancerous tissues from three NSCLC patients, including two males and one female, were obtained from the Department of Thoracic Surgery of Tangdu Hospital, Xi’an, China, and were stored in pre-cooled MACS Tissue Storage Solution (Miltenyi Biotec) at 4 °C immediately after surgical removal. Consistently, these patients were pathologically diagnosed with LUAD and had not undergone chemotherapy or radiotherapy prior to tumor resection. Written informed consent was provided by all patients. The study protocol was approved by the Ethics Committee of the Air Force Military Medical University (KY20226208), and was conducted in accordance with the Declaration of Helsinki.

### NK cell isolation and purification

PBMCs from human peripheral blood samples were isolated using density gradient centrifugation. Following treatment with erythrocyte lysate, NK cells were isolated and purified from the PBMCs using the NK Cell Isolation Kit (Miltenyi Biotec, Cat#130092657). The purity of the enriched NK cells was determined by flow cytometry using a CD3^-^CD56^+^ gating strategy to identify NK cells. The purity of the enriched NK cells exceeded 95%, making them suitable for subsequent experiments.

### Cell culture

The NK92 cell line, a gift from the Department of Pharmacy, Air Force Military Medical University, Xi’an, China, was cultured in α-MEM medium (Gibco, Cat#8122666) supplemented with 12.5% horse serum (Gibco, Cat#1832764), 12.5% fetal bovine serum (FBS, BI, Cat#04-010-1 A), 0.1 mM β-mercaptoethanol (Macklin, Cat#M917637), 100 U/mL IL-2 (PeproTech, Cat#082112), and 1% penicillin-streptomycin (Absin, Cat#abs9244) at 37 °C under 5% CO_2_.

NSCLC cell lines A549 and PC-9, purchased from the Shanghai Cell Bank, Shanghai, China, were cultured in RPMI1640 medium supplemented with 10% FBS and 1% penicillin-streptomycin.

### Single-cell RNA-sequencing

Cells from tumor and paracancerous lung tissue samples were counted using a Countess®II Automated Cell Counter. The proportion of live cells met the following minimum quality control criteria: >90% live cells, ≥1000 cells/μL. Short-read long sequencing and microfluidic techniques were adopted to simultaneously analyze the transcriptome expression profiles of 500–10,000 cells per sample. PCR amplification was performed using cDNA as a template, enabling the construction of a standard sequencing library. High-throughput sequencing of the constructed libraries was carried out in double-ended sequencing mode on the Illumina sequencing platform, using the10x Genomics kit, Chromium single cell 3′ reagent kits (V2).

### Acquisition of NSCLC-conditional medium

NSCLC cell lines (A549 and PC-9) were treated with ER stress inducers (TM, Sigma-Aldrich, Cat#654380, 1.25 μg/mL; TG, Sigma-Aldrich, Cat#T9033, 500 nM) or inhibitors (4-PBA, Sigma-Aldrich, Cat#SML0309, 1 mM; 4μ8C, MCE, Cat#HY-19707, IRE1α pathway-specific inhibitors, 20 μM) for 24 h, washed twice with PBS, and then replaced with fresh medium followed by incubation for 24 h. The supernatants were subsequently collected and centrifuged at 1500 rpm for 5 min. A549 and PC-9 cells were cultured at 1%O_2_, 5%CO_2_, and 37 °C for 24 h to mimic the conditions of NSCLC cells within the TME. NSCLC CM was then used to culture NK92 cells.

### LDH release assay

Cytotoxicity of purified NK cells was determined using the LDH Cytotoxicity Assay Kit (Beyotime, Cat#C0017). NK cells in the simulated tumor microenvironment (TME) were co-cultured with A549 cells in 96-well plates at varying effector-to-target ratios for 8 h at 37 °C. After centrifugation at 400 g/min for 5 min, 120 μL of supernatant from each well was transferred to a new 96-well plate. Then, 60 μL of LDH detection working liquid (prepared according to the manufacturer’s instructions) was added and incubated on a shaker for 30 min in the dark. The absorbance was measured at 490 nm, with the absorbance value at 600 nm used as a control. The resulting absorbance values were substituted into the formula provided in the manual to calculate the killing activity of NK cells.

### Flow cytometry

To analyze surface markers, NK cells were stained in PBS containing 0.1% NaN_3_ and 5% FBS. Surface proteins were stained for 30 min at 4 °C. The following conjugated mouse anti-human antibodies were used for FACS analysis: CD3-PerCP/Cy5.5 (Cat#981008), CD19- PerCP/Cy5.5 (Cat#982412), CD14-PE/Cy7 (Cat#982510), CD66b-FITC (Cat#984102), CD56-APC/Cy7 (Cat#985904), CD16-Pacific Blue (Cat#980106), NKp30-PE/Cy7 (Cat#325214), NKp44-PE (Cat#325108), NKp46-FITC (Cat#331922), NKG2D-APC/Cy7 (Cat#320824), and CD226-APC (Cat#338312), which were purchased from BioLegend. Homologous IgG antibodies conjugated with the same fluorescent dye (Cat#981916, Cat#981816, Cat#984302, Cat#400128, Cat#981812, Cat#981804, Cat#981806) were used as the isotype control. The cells were resuspended in 300 μL of PBS containing 5% FBS. Subsequently, 100,000 cells per sample were collected and analyzed using a standard FACS Calibur flow cytometer (BECKMAN), with the data processed using FlowJo software (TreeStar, RRID:SCR_008520). The gating strategy used to identify NK cells was CD3^−^CD19^−^CD14^−^CD66b^−^CD56^+^.

### Enzyme-linked immunosorbent assay (ELISA)

IL-6 levels in the conditioned medium from NSCLC cell lines were measured using ELISA kits (R&D Systems and Invitrogen, Cat#88-7066). Levels of IL-1β, IL-2, IL-4, IL-6, IL-8, IL-10, IL-12, IL-13, TNF-α, and IFN-γ in the peripheral blood plasma from 11 NSCLC patients and 14 healthy volunteers were assessed using a Luminex bead array (ProcartaPlex, eBioscience) as per the manufacturer’s instructions. Acquisition was performed on a LUMINEX® 100/200™ analyzer (Luminex), and data was analyzed with ProcartaPlex Analyst 1.0 (eBioscience).

### RNA isolation and quantitative PCR

RNA was extracted from cells using TRIzol (Sigma, Cat#BCCH0058). cDNA was synthesized using Hifair® III 1st Strand cDNA Synthesis SuperMix for qPCR (gDNA digester plus) (Yeasen, Cat#11141ES10), and RT-qPCR was performed using qPCR SYBR Green Master Mix (Yeasen, Cat#11184ES03) on a Step One Real-time PCR instrument (Roche, Switzerland), according to the manufacturer’s protocol. Relative gene expression was analyzed using the 2^–ΔΔCt^ method, normalized to GAPDH and appropriate controls. The primers used are listed in Table S1.

### Luciferase reporter assay

Wild-type (WT) or truncated constructs of promoter fragments of the indicated genes were cloned upstream of the firefly luciferase reporter in a pGL4.10 vector. The indicated cells were co-transfected with the respective pGL4-promoter plasmid, a pRL-SV40 Renilla luciferase reporter plasmid, and an expression vector encoding the indicated transcription factor or a control vector. Firefly and Renilla luciferase activities were measured using the Dual-Luciferase Reporter Assay System (Tecan). Firefly luciferase activity was normalized to Renilla luciferase activity and presented as relative luciferase activity.

### Immunofluorescence

The human NSCLC tumor tissues and adjacent tissues were embedded in paraffin and sectioned into 3 µm thick slices. The slices were incubated at 65 °C for 2 h, dewaxed using a gradient ethanol series, and subjected to antigen retrieval with EDTA 9.0 repair solution. Blocking was performed with 5% BSA for 1 h at room temperature. Primary antibodies, including anti-human IRE1α (CST, 3294, 1:100), anti-human XBP1s (CST, 40435, 1:100), anti-human UBE2S (ImmunoWay, YM3636, 1:100), and anti-human NKp30 (ImmunoWay, YT3135, 1:100), were added and incubated overnight at 4 °C. The sections were then incubated with fluorescent secondary antibodies for 1 h at room temperature and rinsed thrice with PBST. Nuclei were stained with DAPI for 5 min in the dark, after which the sections were sealed with an anti-fluorescence quenching sealer and examined under a confocal microscope.

### Cell transfection

NK92 were cultured in six-well plates in α-MEM medium without penicillin/streptomycin for 8 h. A total of 7.5 μL of siRNA was diluted in 200 μL of α-MEM medium (Gibco), mixed well, and left to stand for 5 min at room temperature (RT). Separately, 7.5 μL of Lipofectamine RNAiMAX (Invitrogen, 13778-150) was dissolved in 200 µL α-MEM medium and incubated for 5 min at RT. The above two solutions were then combined and allowed to incubate for 10–15 min at RT to form the transfection mixture. Following centrifugation, the original medium was discarded, and 1600 µL of fresh medium along with 400 µL of the siRNA transfection mixture were added to the cells. The cells were incubated for 6 h in a cell incubator. Subsequently, the transfection medium was replaced with cell growth medium, and the cells were cultured for another 48 h for subsequent experiments.

### Co-immunoprecipitation (CoIP)

The indicated NK92 cells were lysed using Pierce IP Lysis Buffer (Thermo Fisher Scientific, Cat#88804) supplemented with protease and phosphatase inhibitor cocktail, without cross-linking. NKp30 antibody was co-incubated with lysates from various treated NK cell at 4 °C overnight with constant rotation. Rabbit IgG was used as a negative control. The antigen sample/antibody mixture was then transferred to a centrifuge tube containing magnetic beads and incubated for 1 h at room temperature with constant rotation. The proteins bound to the magnetic beads were eluted under low pH conditions, boiled with SDS loading buffer, and subsequently analyzed by Western blot.

### Statistical analysis

Data from three independent experiments were presented as mean ± SEM. Unpaired Student’s t-test and one-way analysis of variance (ANOVA) were used depending on the type of experiment. A *P* value < 0.05 was considered to be statistically significant. All analyses were performed using GraphPad Prism 8 software (GraphPad Software, RRID:SCR_002798).

## Supplementary information

Supplementary Information

Supplementary Information

## Data Availability

The datasets generated during and/or analyzed during the current study are available from the corresponding author on reasonable request.
