# Supplementary material for: Mechanistic insights into IL-6-mediated NK cell dysfunction in NSCLC via the IRE1α-XBP1s-STAT3-UBE2S axis
Source: NPJ Precis Oncol. 2025 Nov 18;9:361. doi: 10.1038/s41698-025-01140-z (PMC12627650; doi:10.1038/s41698-025-01140-z)
Supplement: Supplementary file 1 — Supplementary Information [file 41698_2025_1140_MOESM1_ESM.docx]

**Supplementary Materials**

**
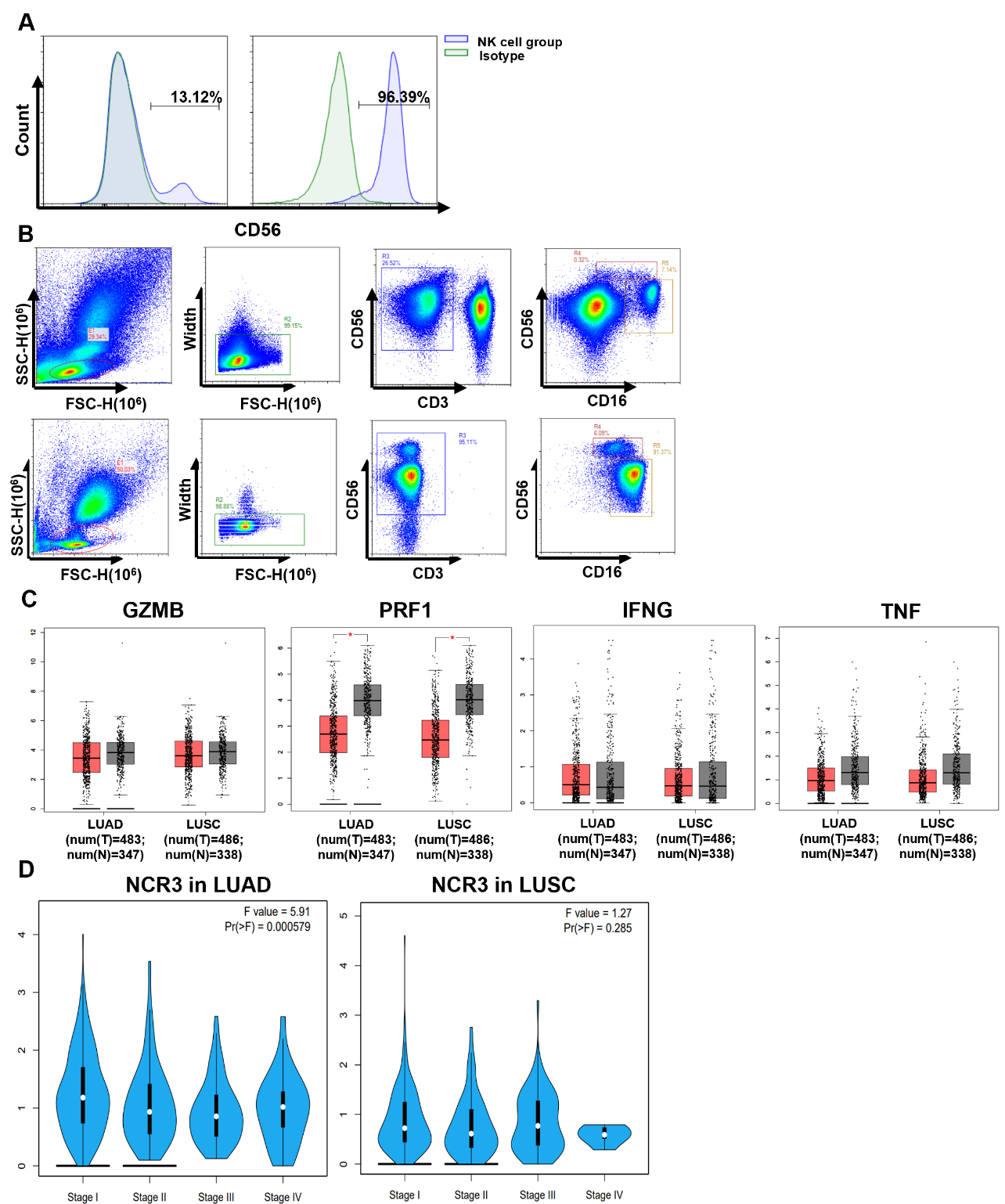
Supplementary Figures**

**Figure S1. The functional characteristics and purification process of NK cells in patients with NSCLC**

(A) Flow cytometry was used to detect the purity of NK cells after magnetic bead negative sorting. (B) Comparison of peripheral blood NK cell purity before and after magnetic bead negative sorting and ring gate strategy. (C) The results of the database showed the expression of factors related to NK cell function in patients with lung adenocarcinoma (LUAD) and lung squamous cell carcinoma (LUSC). (D) NCR3 expression in NSCLC tissues from patients stratiﬁed by pathological characteristics or stage.


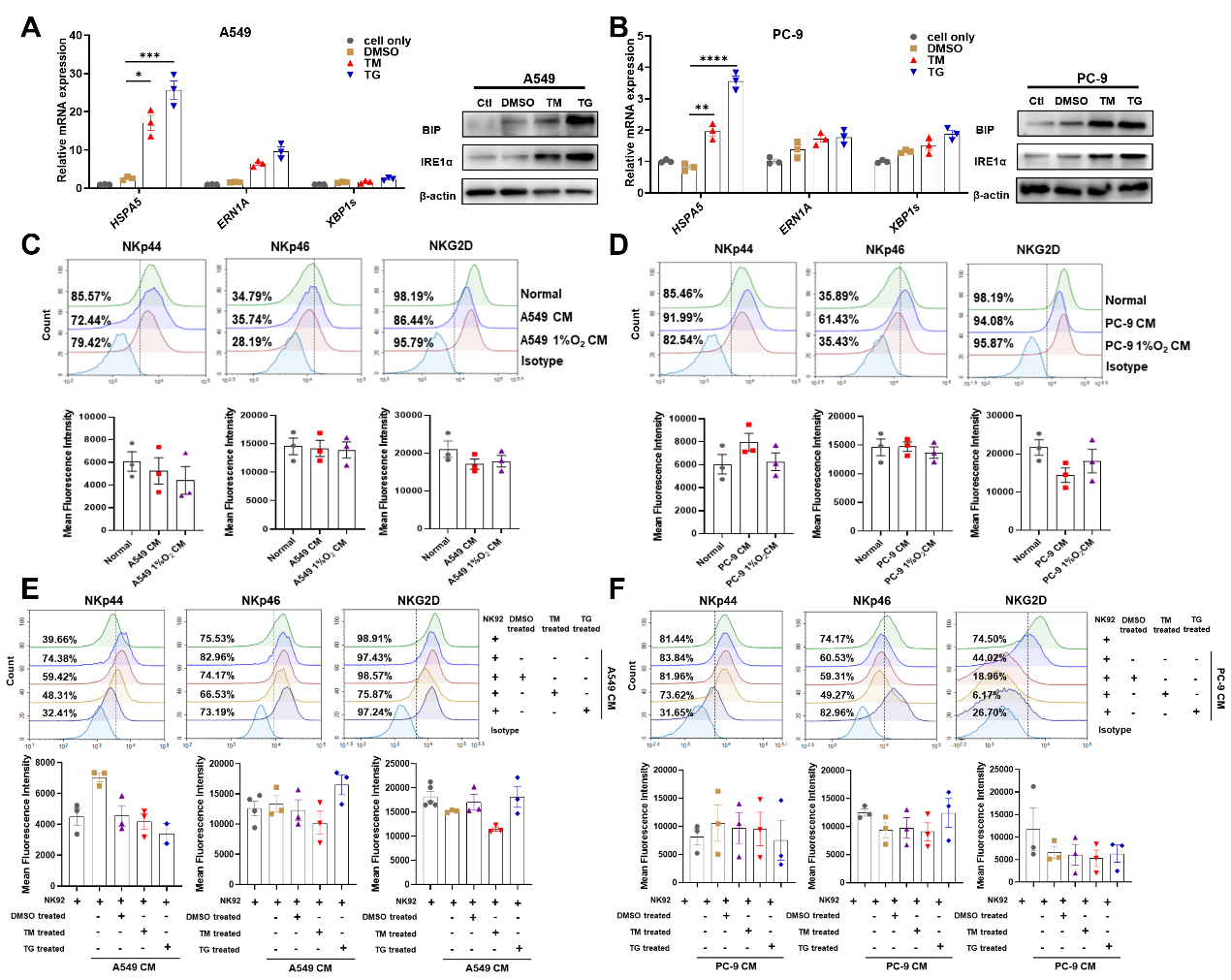


**Figure S2. Detection of UPR status of NSCLC cells after different treatments and its effect of CM on NK cell activating receptor expression**

(A) HSPA5, ERN1 and XBP1s expression levels in A549 cells treated with DMSO, TM or TG. Representative Western blot images for BIP in A549 cells treated with DMSO, TM or TG. (B) HSPA5, ERN1 and XBP1s expression levels in PC-9 cells treated with DMSO, TM or TG. Representative Western blot images for BIP in NK92 cells treated with DMSO, TM or TG. (C) Representative histograms and mean fluorescence intensity values corresponding to NK cell activating receptor expression by NK-92 cells treated with CM from A549 cells cultured under hypoxic (1% O2) or normoxic conditions. (D) Representative histograms and mean fluorescence intensity values corresponding to NK cell activating receptor expression by NK-92 cells treated with CM from PC-9 cells cultured under hypoxic (1% O2) or normoxic conditions. (E) Representative histograms and mean fluorescence intensity values corresponding to NK cell activating receptor expression by NK-92 cells treated with A549 TM CM or TG CM. (F) Representative histograms and mean fluorescence intensity values corresponding to NK cell activating receptor expression by NK-92 cells treated with PC-9 TM CM or TG CM. Data were analyzed by one-way ANOVA. **p* < 0.05, ***p* < 0.01, ****p* < 0.001, *****p* < 0.0001.


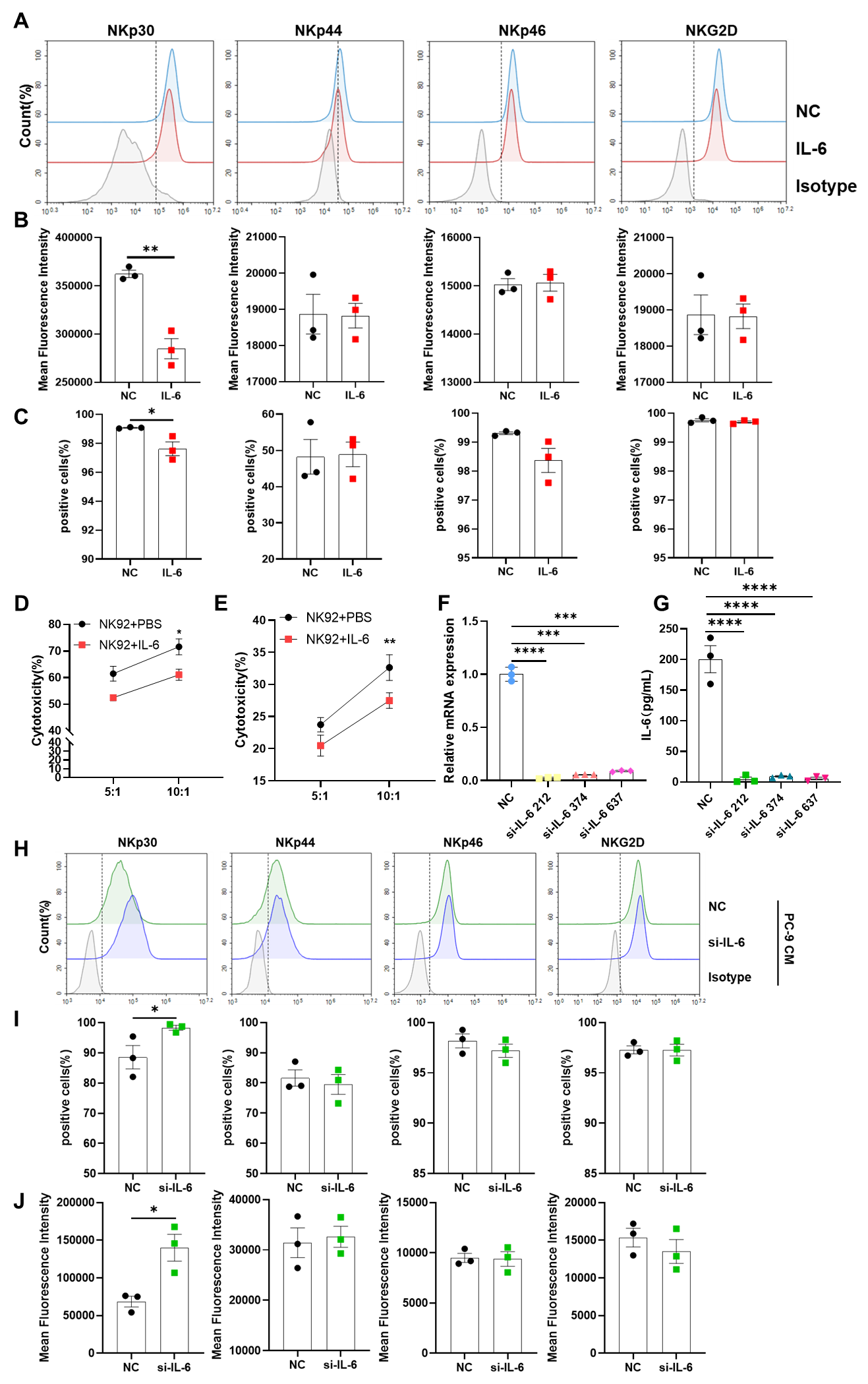


**Figure S3. IL-6 inhibits NKp30 expression and impairs NK cell function**

(A-C) Representative histograms and mean fluorescence intensity values corresponding to activating receptor expression in NK-92 cells treated with recombinant IL-6 (100 pg/mL) for 24 h (n=3). (D, E) LDH release assay was used to evaluate the effect of IL-6 on the cytotoxic activity of NK92 cells against A549 (D) and PC-9 (E) cell lines (n=3). (F) Alterations in IL-6 mRNA expression level following IL-6 knockdown in PC-9 cells. (G) Alterations in IL-6 protein secretion level following IL-6 knockdown in PC-9 cells. (H-J) Representative histograms and mean fluorescence intensity measurements depicting the expression of activating receptors in NK-92 cells following a 24-hour treatment with conditioned medium obtained from PC-9 cells subjected to siRNA treatment or left untreated (n=3). Data were analyzed by one-way ANOVA. **p* < 0.05, ***p* < 0.01.


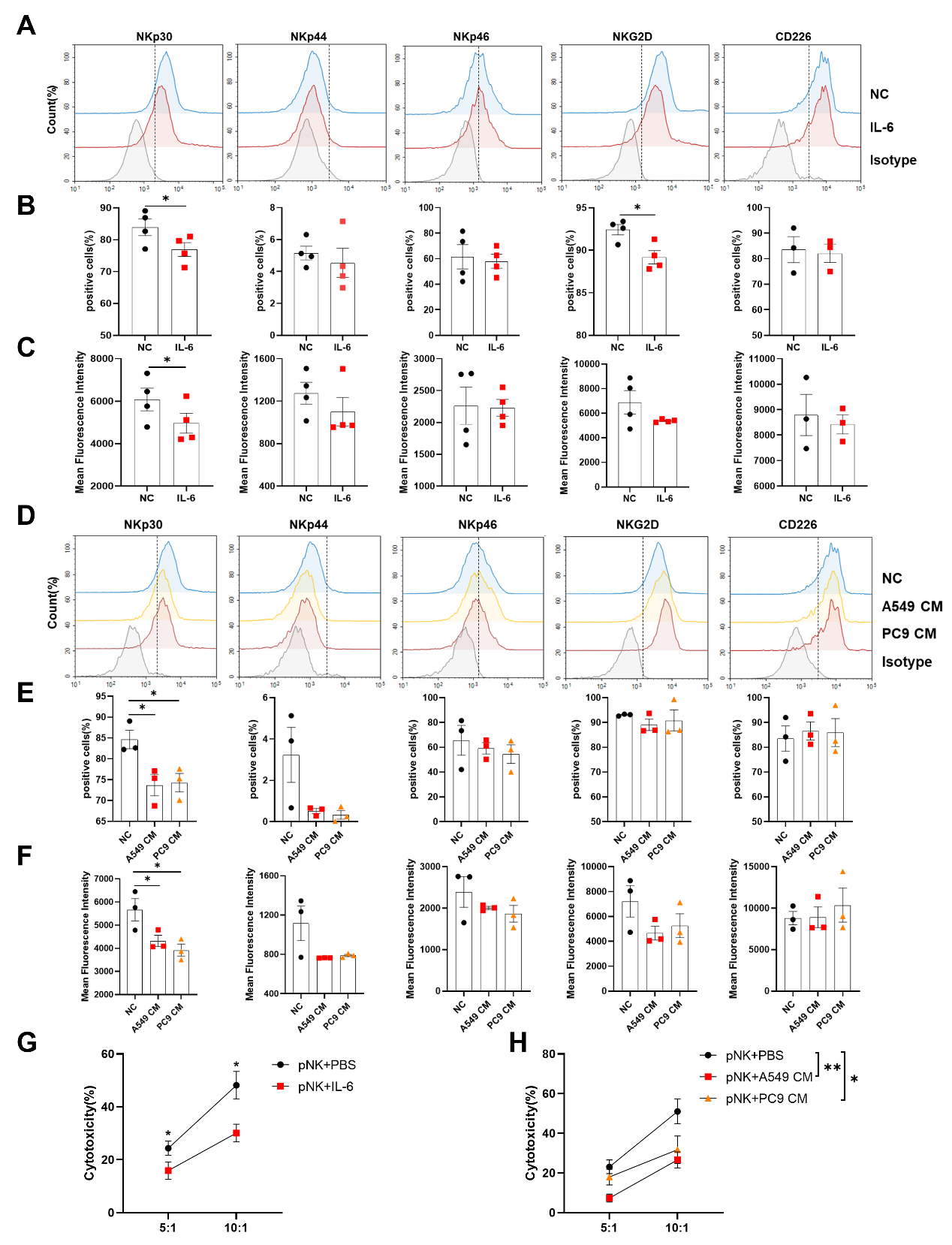


**Figure S4. IL-6 inhibits NKp30 expression and impairs peripheral blood NK cell function**

(A-C) Representative histograms and mean fluorescence intensity values corresponding to activating receptor expression in peripheral blood NK cells treated with recombinant IL-6 (100 pg/mL) for 24 h (n=3). (D-F) Representative histograms and mean fluorescence intensity measurements depicting the expression of activating receptors in peripheral blood NK cells following a 24-hour treatment with conditioned medium obtained from PC-9 cells or A549 cells (n=3). (G) LDH release assay was used to evaluate the effect of IL-6 on the cytotoxic activity of peripheral blood NK cells (n=3). (H) LDH release assay was used to evaluate the effect of conditioned medium on the cytotoxic activity of peripheral blood NK cells (n=3). CM: conditioned medium; pNK: peripheral blood NK cell. Data were analyzed by one-way ANOVA. **p* < 0.05, ***p* < 0.01.


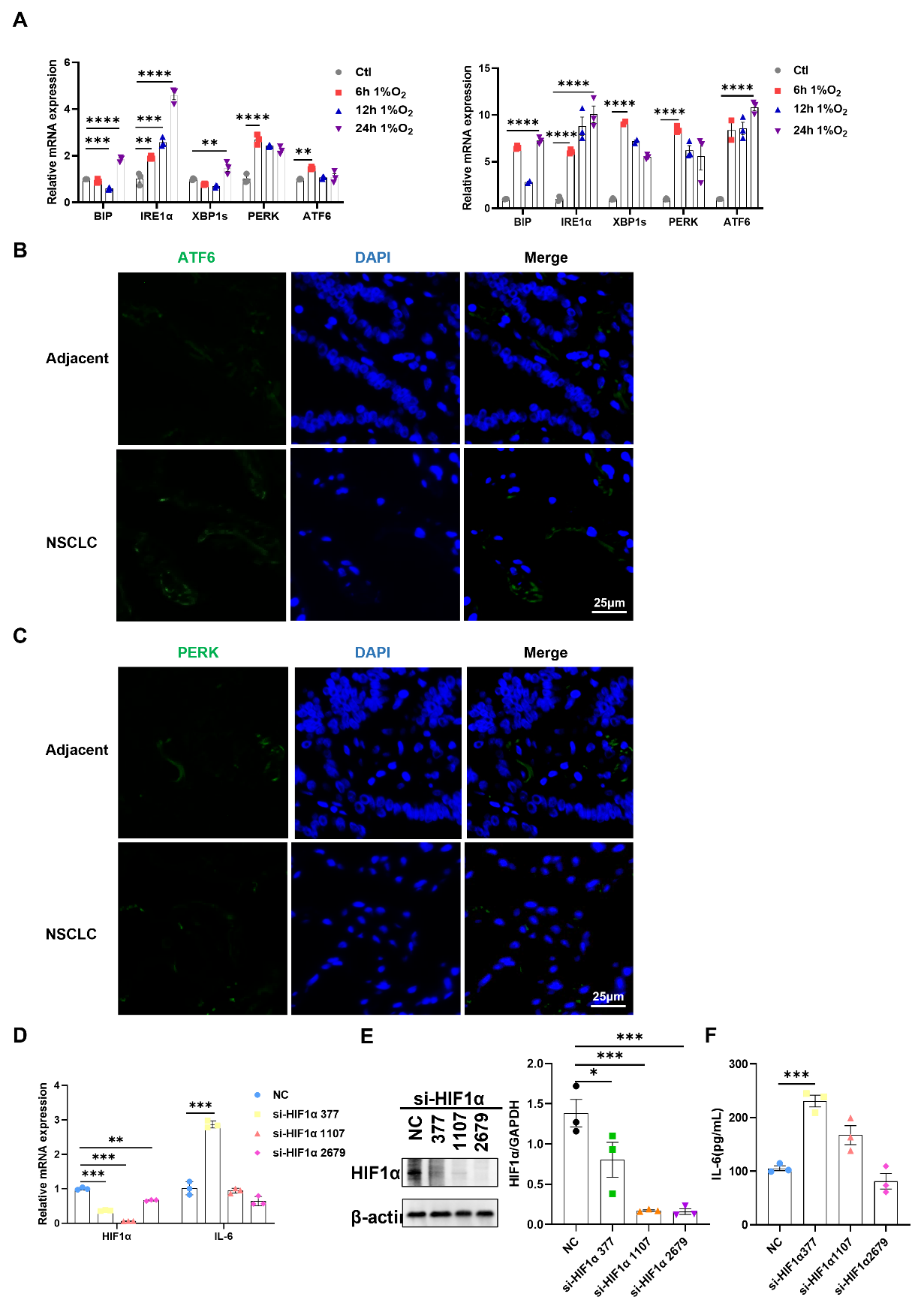


**Figure S5.** **Detection of UPR marker molecule expression in NSCLC cells under in vitro and in vivo conditions.**

(A) Transcript levels of UPR-related genes in NSCLC cells incubated for different times in hypoxia. (B, C) Expression levels of UPR marker molecules in NSCLC tumor tissues and paracancerous tissues. (D) Transcript levels of HIF1α and IL-6 in NSCLC cells cultured in hypoxia for 24 h after siRNA knockdown of HIF1α. (E) Representative Western blot images for HIF1α in NSCLC cells cultured in hypoxia for 24 h after siRNA knockdown of HIF1α. (F) IL6 expression level in NSCLC cells cultured in hypoxia for 24 h after siRNA knockdown of HIF1α (n=3). Data were analyzed by Student’s t test and one-way ANOVA. * *p* < 0.05, ** *p* < 0.01, *** *p* < 0.001.


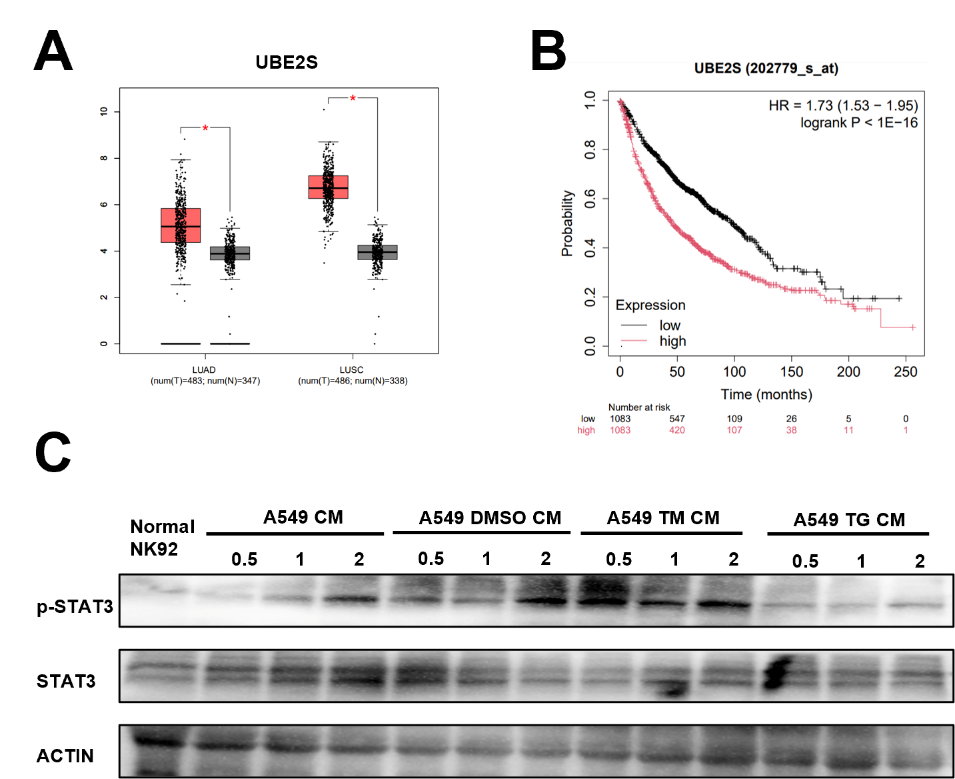


**Figure S6. Detection of expression levels of IL-6 downstream-related pathway molecules in NK cells after treatment with different CM.**

(A) UBE2S expression in NSCLC tissues from patients stratiﬁed by pathological characteristics. (B) Kaplan-Meier survival of NSCLC patients based on UBE2SmRNA level (UBE2S low, black; UBE2S high, red). (C) Phosphorylation levels of STAT3 in NK cells after treatment with different CM for different times.


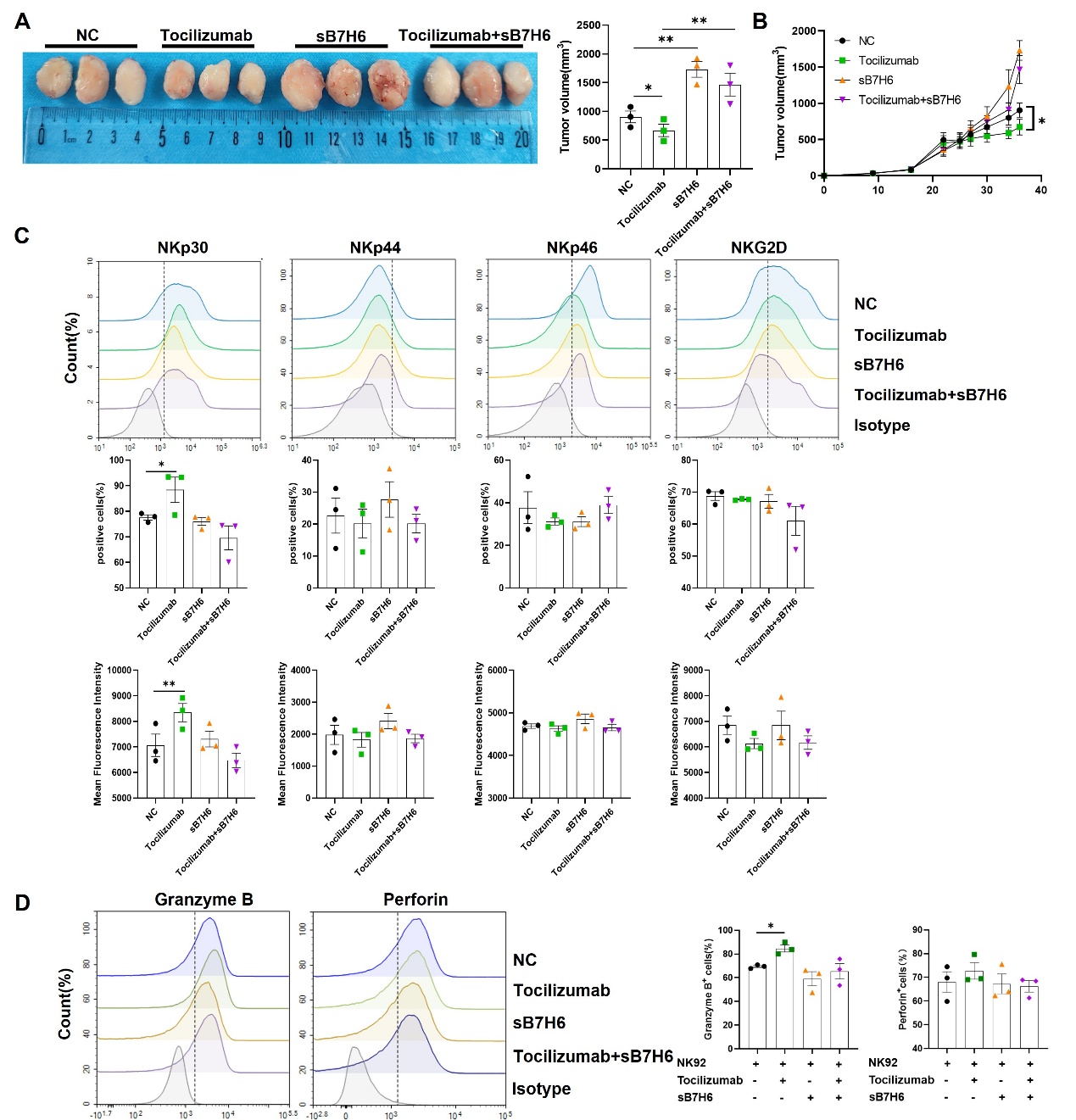


**Figure S7.** **Targeting STAT3/UBE2S/NKp30 Enhances NK Cell Function and Suppresses NSCLC**

(A) Representative images of tumors in PC-9 cell-derived C-NKG mouse subcutaneous tumor models across various treatment groups, accompanied by corresponding tumor volume measurements. (B) Growth curve of tumors derived from PC-9 cells in the C-NKG mouse subcutaneous tumor model across various treatment groups. (C) Representative flow cytometry plots and corresponding statistical analyses depicting the expression levels of surface activation receptors on NK92 cells infiltrating tumors following three intravenous administrations of 1 × 10^7^ NK92 cells. (D) Representative flow cytometry plots and corresponding statistical analyses depicting the expression levels of granzyme B and perforin secreted by tumor-infiltrating NK92 cells. NC: Normal NK92 treatment group; Tocilizumab: The group subjected to tail vein injection was administered a suspension of NK92 cells supplemented with 100 μg/mL Tocilizumab; sB7H6: Treatment group with NK92 cell suspension containing 100 μg/mL soluble B7H6; Tocilizumab+sB7H6: Treatment group with NK92 cell suspension containing 100 μg/mL Tocilizumab and 100 μg/mL soluble B7H6. Treatments were delivered once weekly for three consecutive weeks. Each group comprised three mice with comparable body weights, and no significant differences were observed in either age or body weight across the groups. Data were analyzed by one-way ANOVA. **p* < 0.05, ***p* < 0.01.

**Table S1.** **The primers used in this article.**

| Gene | Forward（5’-3’） | Reverse（5’-3’） |
| --- | --- | --- |
| *HSPA5* | GGTGAAAGACCCCTGACAAA | GTCAGGCGATTCTGGTCATT |
| *ERN1* | AGTATGTGGAGCAGAAGGAC | GTTGTGTGGCTTTAGGTCTC |
| *XBP1s* | GCAGGTGCAGGCCCAGTTGT | TGGGTCCAAGTTGTCCAGAATGC |
| *EIF2AK3* | GTCCGGAACCAGACGATGAG | GGCTGGATGACACCAAGGAA |
| *ATF6* | CAGCAGCACCCAAGACTCAAACAA | ACCACAGTAGGCTGAGACAGCAAA |
| *DDIT3* | CTGCTTCTCTGGCTTGGCTG | GCTCTGGGAGGTGCTTGTGA |
| *NCR3* | CCCACTTGCTTCTTCCCGTTTCC | CACCACCAGCCGAGTCCCATTCC |
| *IL-6* | CCACTCACCTCTTCAGAACG | CATCTTTGGAAGGTTCAGGTTG |
| *UBE2S* | CGATGGCATCAAGGTCTTTCCC | CAGCAGGAGTTTCATGCGGAAC |
| *GAPDH* | GCCATCAATGACCCCTTCATT | TTGACGGTGCCATGGAATTT |
