# Supplementary material for: Mechanistic insights into IL-6-mediated NK cell dysfunction in NSCLC via the IRE1α-XBP1s-STAT3-UBE2S axis
Source: NPJ Precis Oncol. 2025 Nov 18;9:361. doi: 10.1038/s41698-025-01140-z (PMC12627650; doi:10.1038/s41698-025-01140-z)

unprocessed images

Fig. 5D

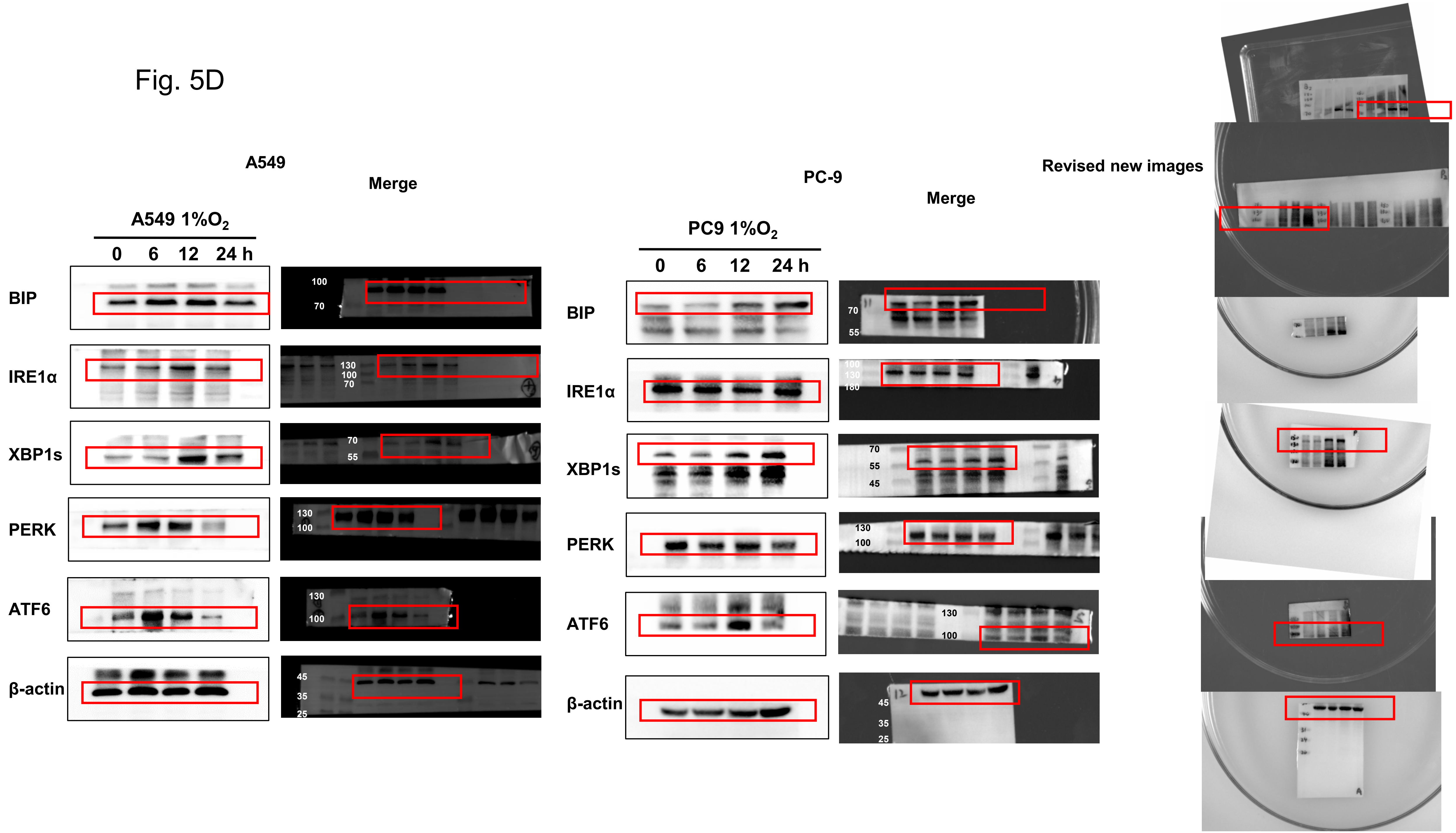

A549
Merge
A549 1%O2
0 6 12 24 h
BIP
IRE1α
XBP1s
PERK
ATF6
β-actin
100
70
130
100
70
70
55
130
100
130
100
45
35
25
PC-9
Merge
PC9 1%O2
0 6 12 24 h
BIP
IRE1α
XBP1s
PERK
ATF6
β-actin
70
55
100
130
180
70
55
45
130
100
130
100
45
35
25
Revised new images

Fig. 6E-F

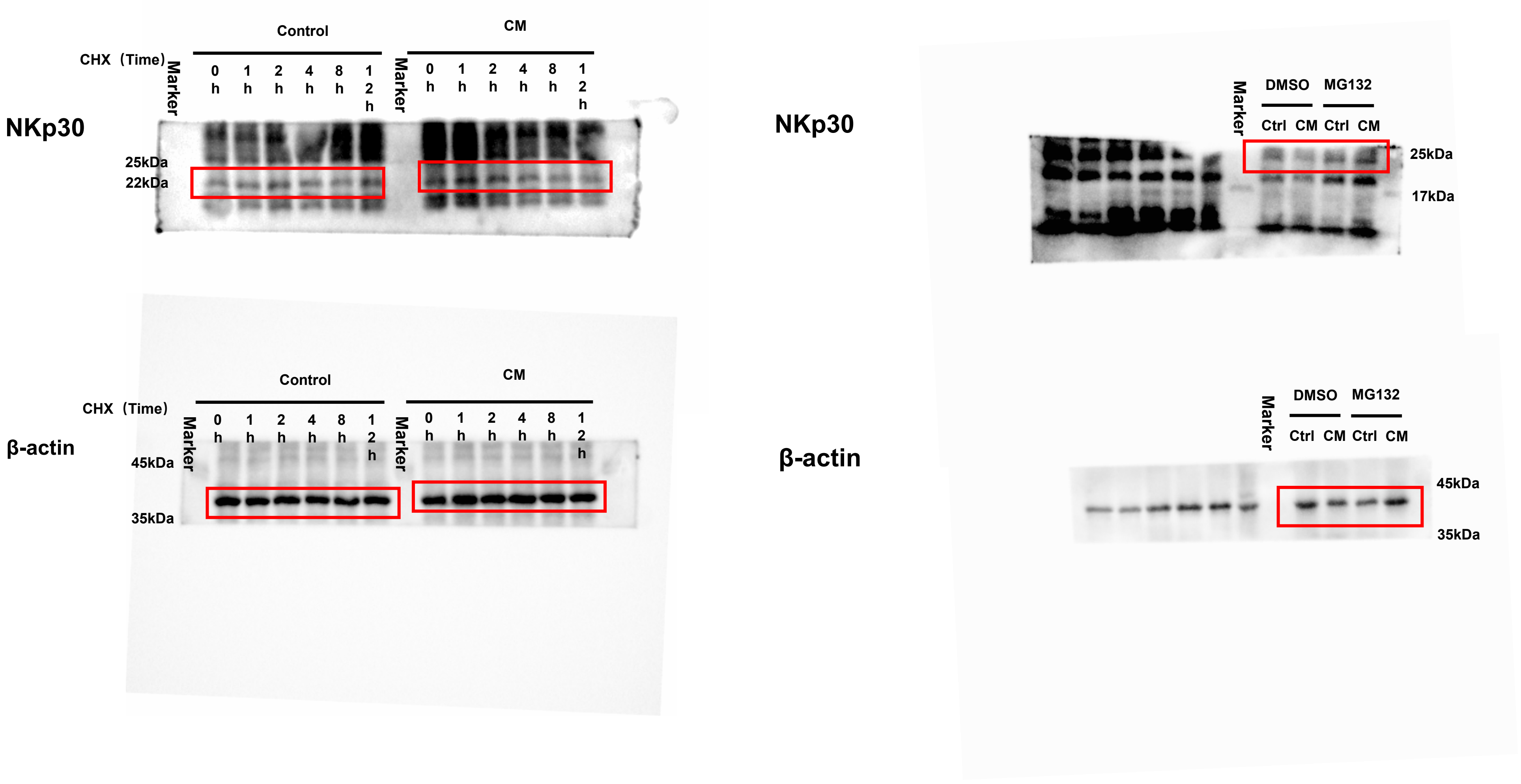

NKp30
Control
CM
CHX（Time）
Marker
0 h
1 h
2 h
4 h
8 h
12 h
25kDa
22kDa
β-actin
45kDa
35kDa
DMSO
MG132
Ctrl
CM
17kDa

Fig. 7C、E

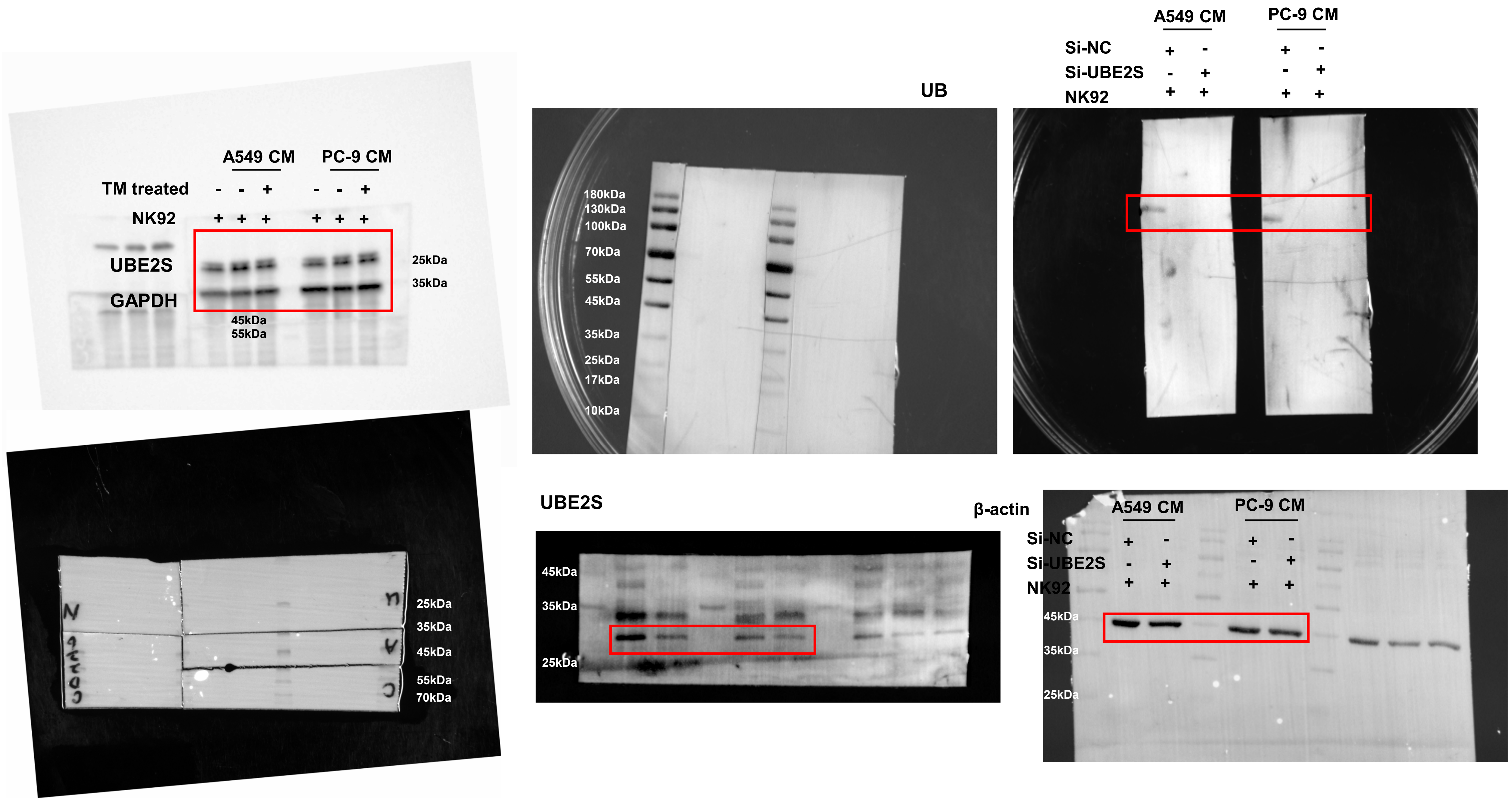
A549 CM
PC-9 CM
TM treated - - + - - +
NK92 + + + + + +
UBE2S
GAPDH
25kDa
35kDa
45kDa
55kDa
70kDa
UB
180kDa
130kDa
100kDa
70kDa
55kDa
45kDa
35kDa
25kDa
17kDa
10kDa
Si-NC + - + -
Si-UBE2S - + - +
NK92 + + + +
UBE2S
β-actin

Fig. 7F

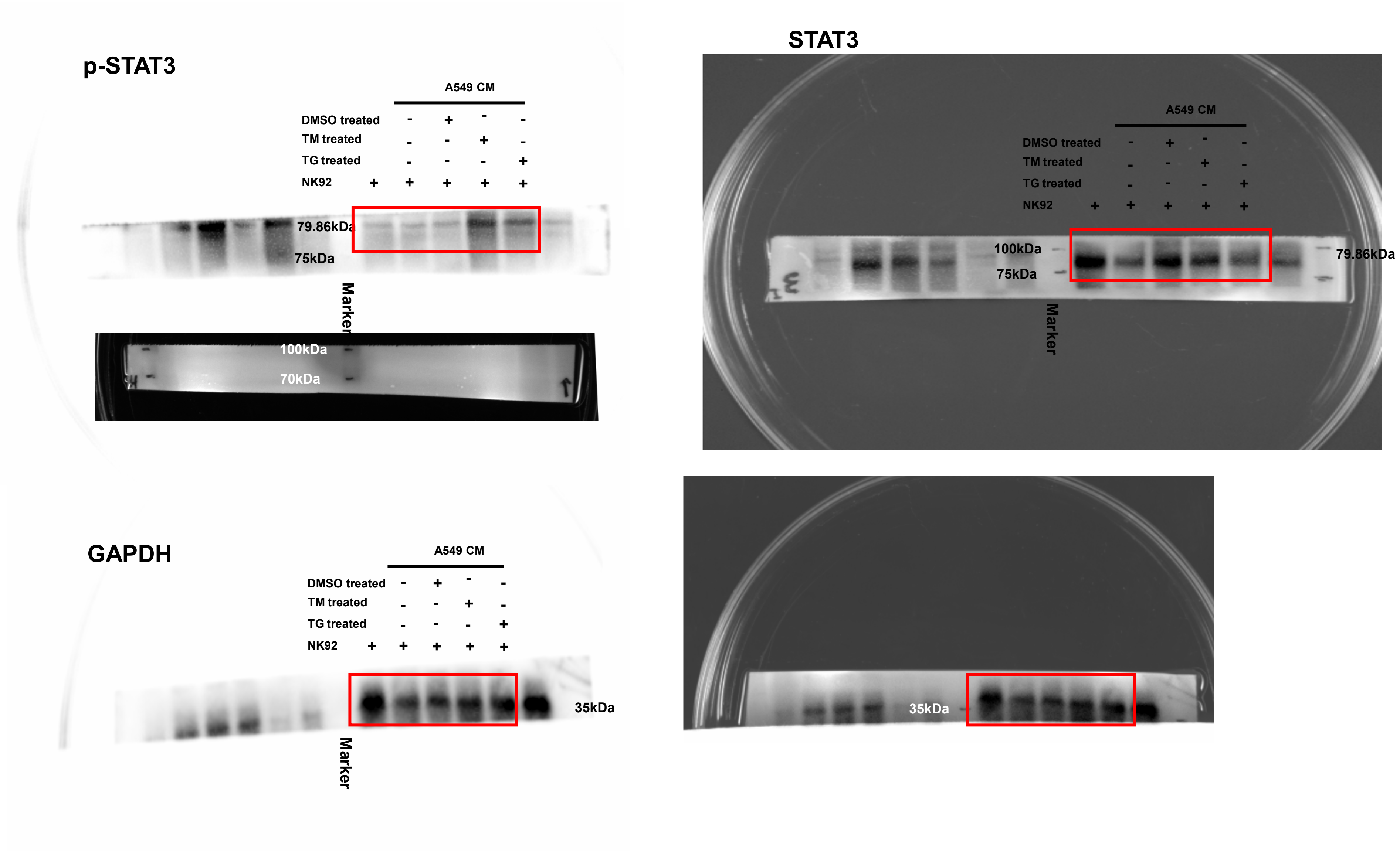
p-STAT3
A549 CM
DMSO treated - + - -
TM treated - - + -
TG treated - - - +
NK92 + + + + +
79.86kDa
75kDa
Marker
100kDa
70kDa
STAT3
A549 CM
DMSO treated - + - -
TM treated - - + -
TG treated - - - +
NK92 + + + + +
100kDa
75kDa
79.86kDa
Marker
GAPDH
A549 CM
DMSO treated - + - -
TM treated - - + -
TG treated - - - +
NK92 + + + + +
35kDa
Marker
35kDa

Fig. 7F

Revised new images

p-STAT3

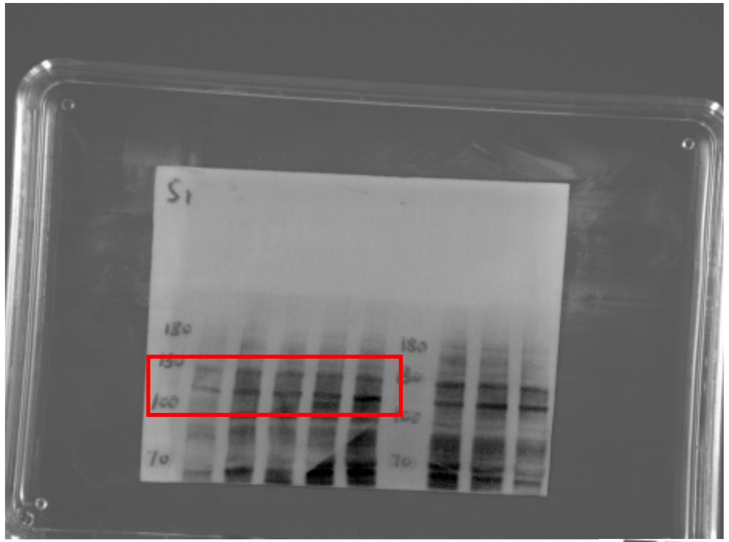

STAT3

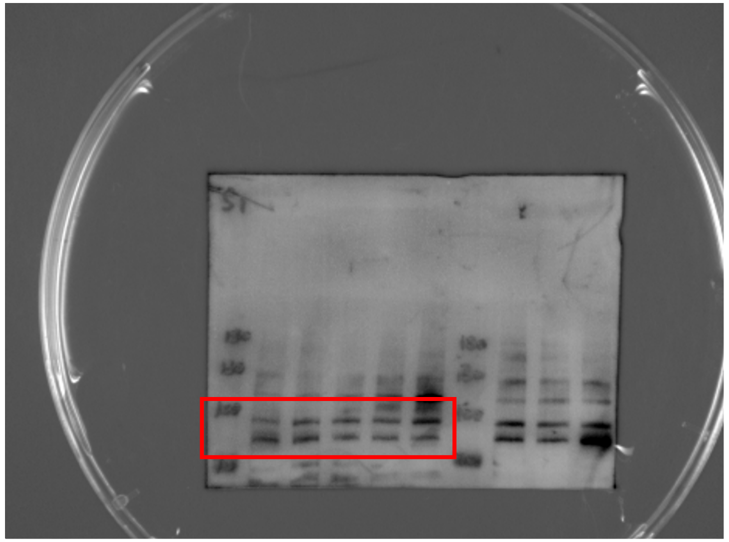

β-actin

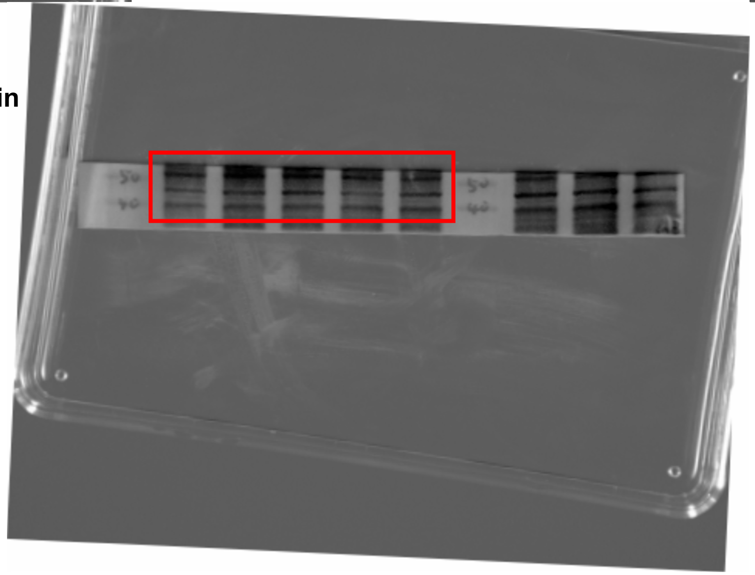

Fig. 7G

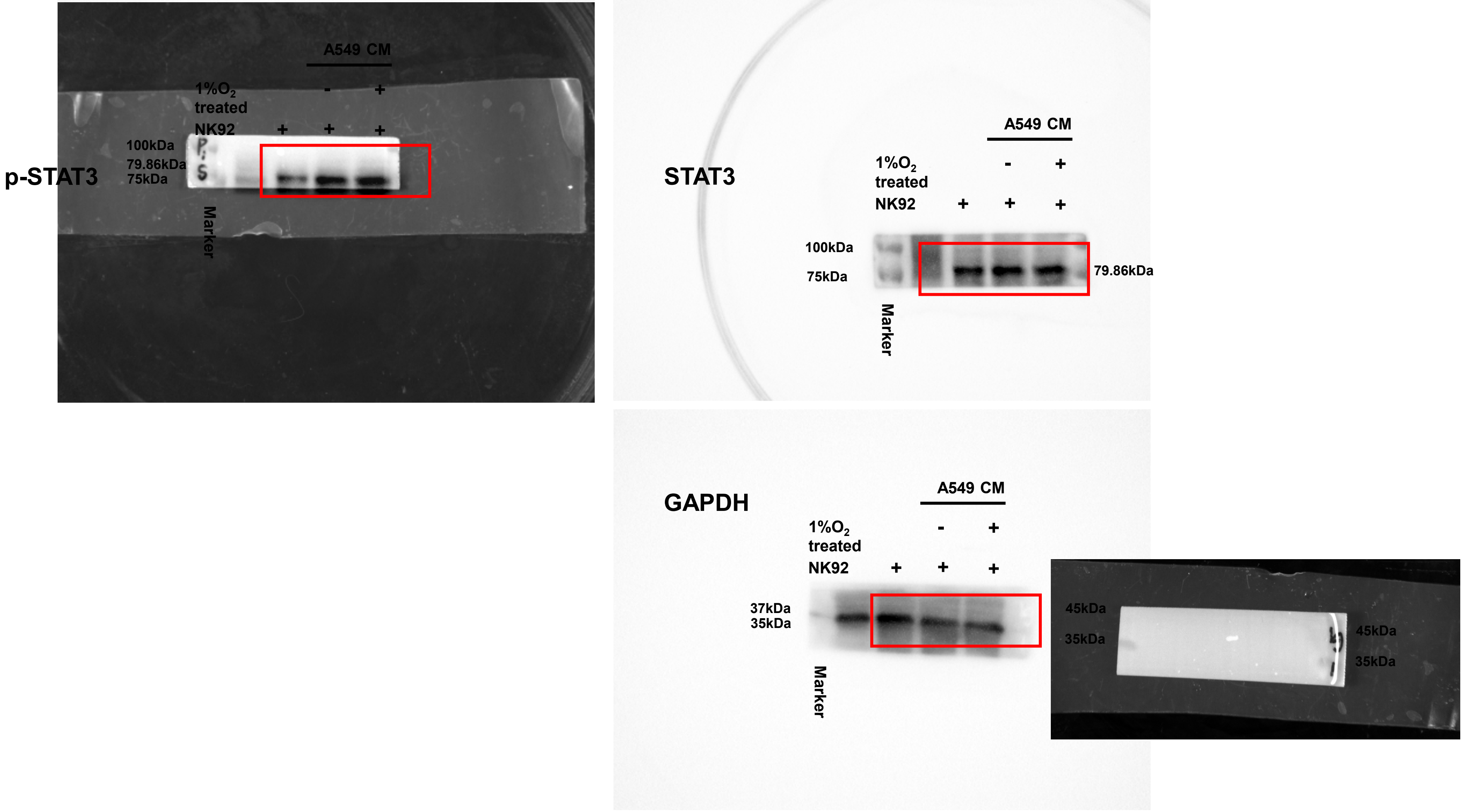
p-STAT3
A549 CM
1%O2 treated
- +
NK92
+ + +
100kDa
79.86kDa
75kDa
Marker
STAT3
A549 CM
1%O2 treated
- +
NK92
+ + +
100kDa
75kDa
79.86kDa
Marker
GAPDH
A549 CM
1%O2 treated
- +
NK92
+ + +
37kDa
35kDa
Marker
45kDa
35kDa
45kDa
35kDa

Fig. 7H

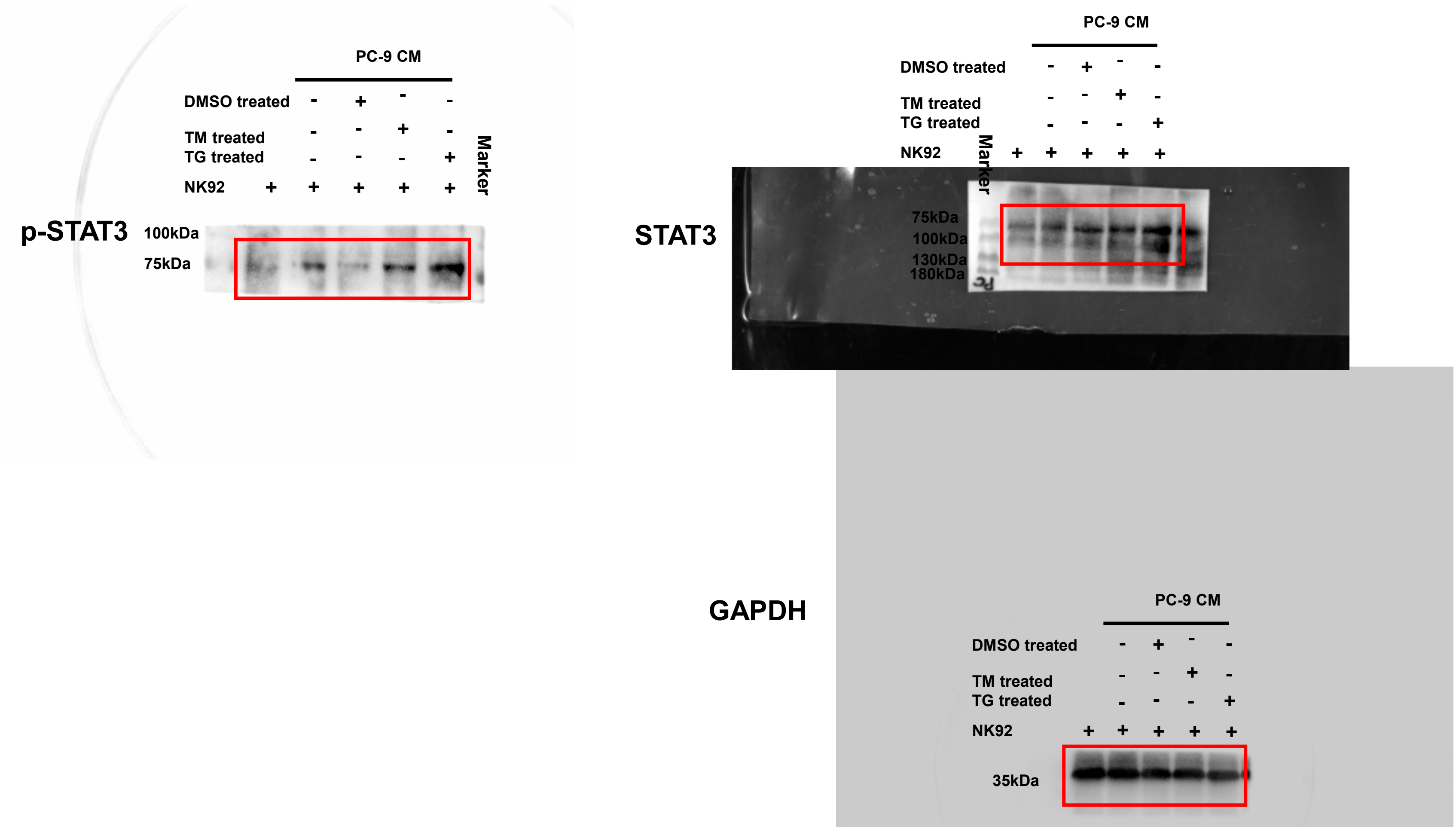

p-STAT3
PC-9 CM
DMSO treated - + - -
TM treated - - + -
TG treated - - - +
NK92 + + + + +
Marker
100kDa
75kDa
STAT3
PC-9 CM
DMSO treated - + - -
TM treated - - + -
TG treated - - - +
NK92 + + + + +
Marker
75kDa
100kDa
130kDa
180kDa
GAPDH
PC-9 CM
DMSO treated - + - -
TM treated - - + -
TG treated - - - +
NK92 + + + + +
35kDa

Fig. 7I

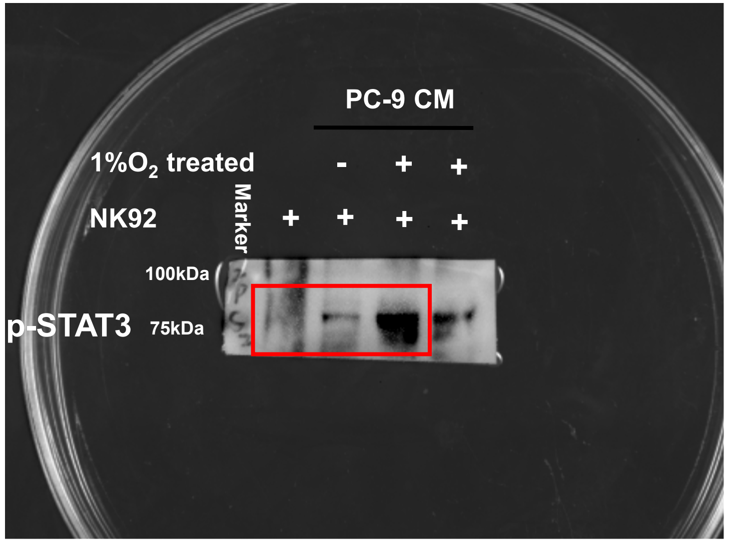

PC-9 CM
$1\%O_2$ treated
-
+
+
NK92
Marker
+
+
+
+
100kDa
p-STAT3
75kDa

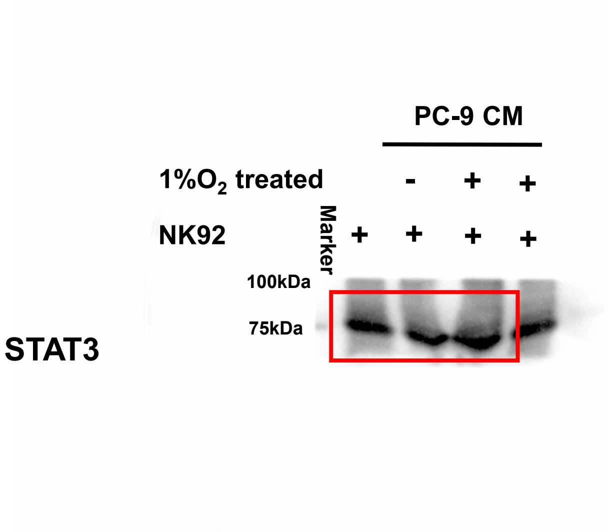

PC-9 CM
$1\%O_2$ treated
-
+
+
NK92
Marker
+
+
+
+
100kDa
75kDa
STAT3

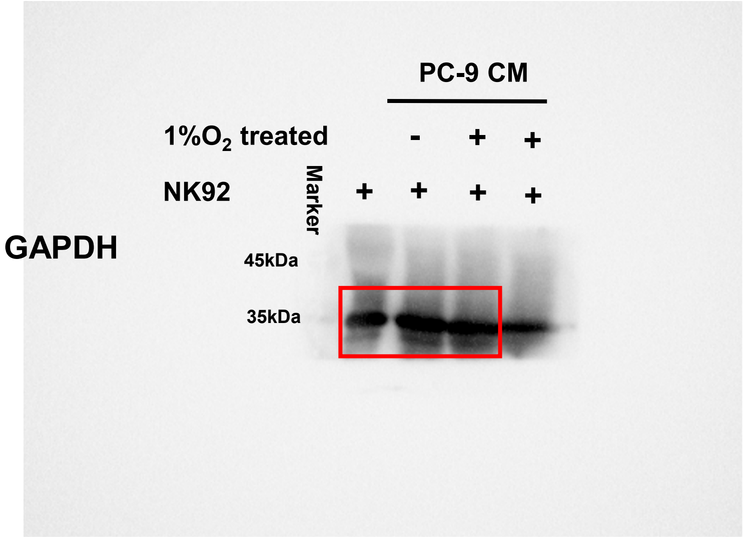

PC-9 CM
$1\%O_2$ treated
-
+
+
NK92
Marker
+
+
+
+
GAPDH
45kDa
35kDa

Fig. 7J

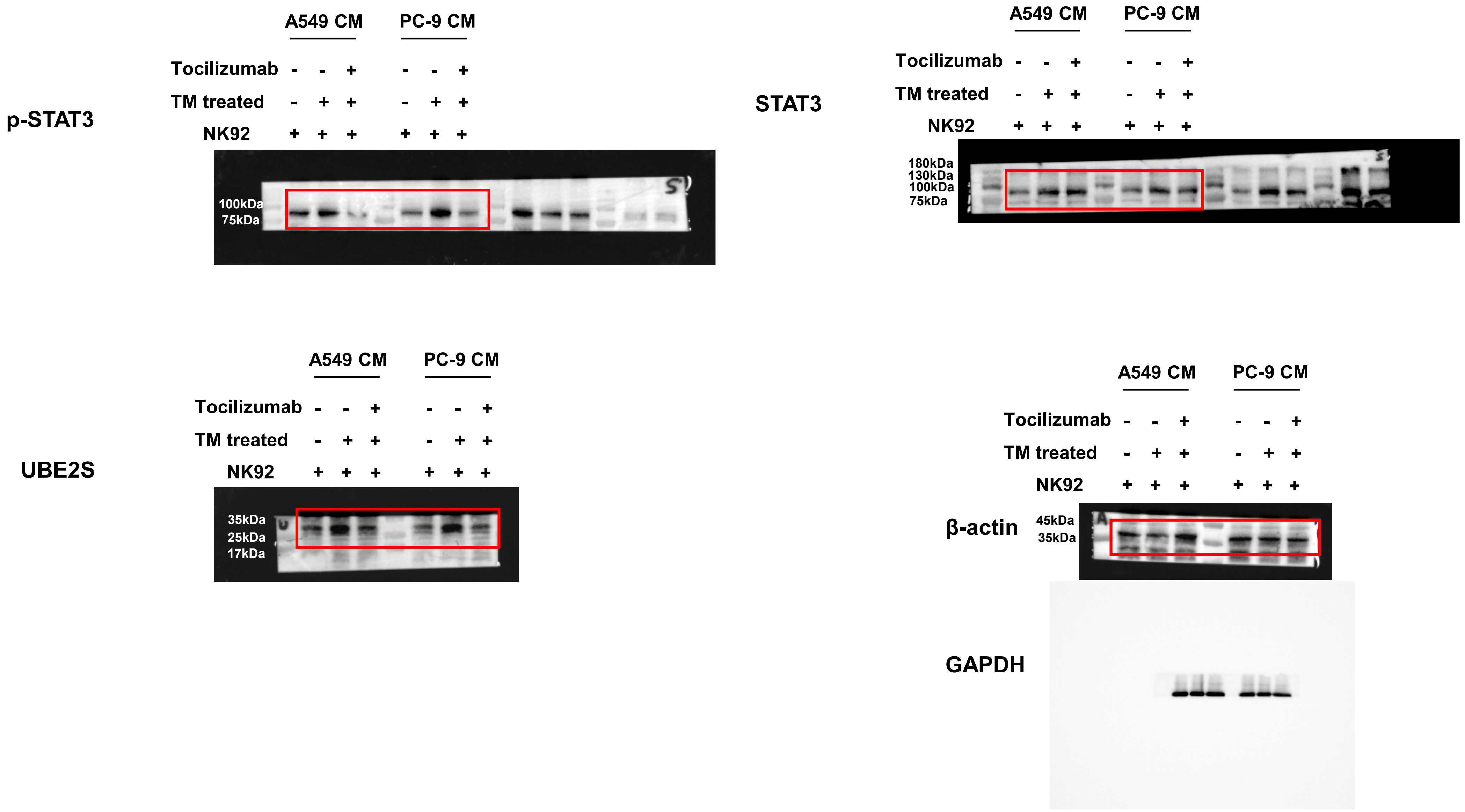

p-STAT3
A549 CM
PC-9 CM
Tocilizumab - - + - - +
TM treated - + + - + +
NK92 + + + + + +
100kDa
75kDa
STAT3
A549 CM
PC-9 CM
Tocilizumab - - + - - +
TM treated - + + - + +
NK92 + + + + + +
180kDa
130kDa
100kDa
75kDa
UBE2S
A549 CM
PC-9 CM
Tocilizumab - - + - - +
TM treated - + + - + +
NK92 + + + + + +
35kDa
25kDa
17kDa
β-actin
A549 CM
PC-9 CM
Tocilizumab - - + - - +
TM treated - + + - + +
NK92 + + + + + +
45kDa
35kDa
GAPDH

Fig. 7K

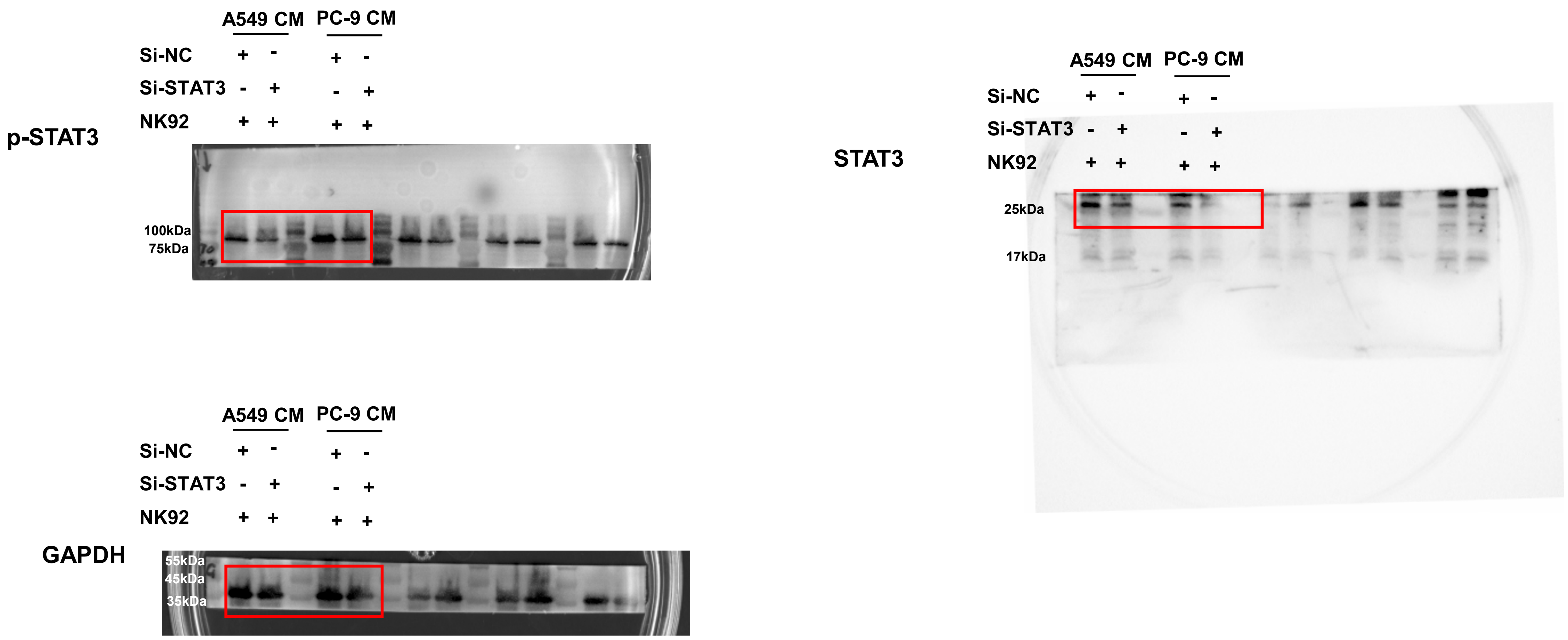
p-STAT3
A549 CM
PC-9 CM
Si-NC + - + -
Si-STAT3 - + - +
NK92 + + + +
100kDa
75kDa
GAPDH
A549 CM
PC-9 CM
Si-NC + - + -
Si-STAT3 - + - +
NK92 + + + +
55kDa
45kDa
35kDa
STAT3
A549 CM
PC-9 CM
Si-NC + - + -
Si-STAT3 - + - +
NK92 + + + +
25kDa
17kDa

Supplementary Fig. 2A、B

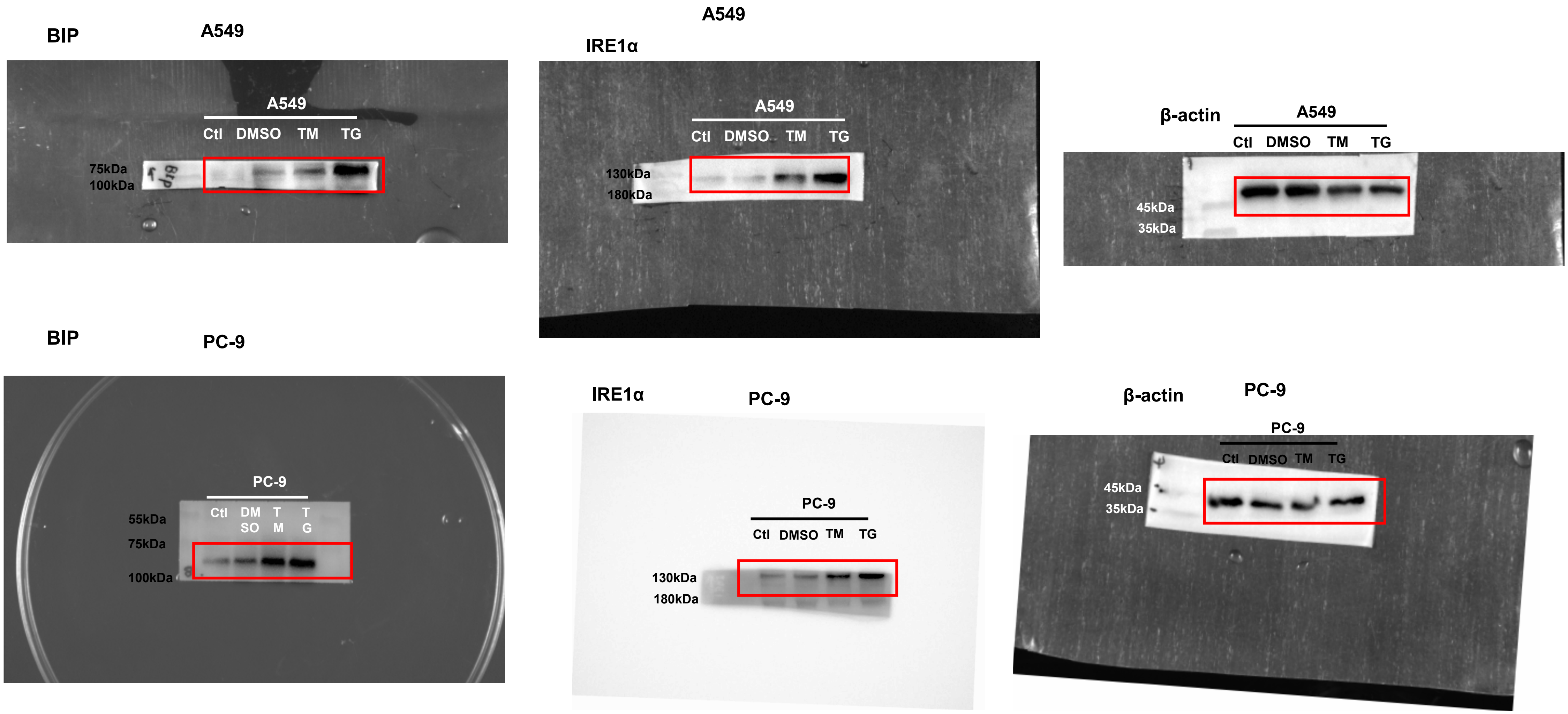

Supplementary Fig. 5E

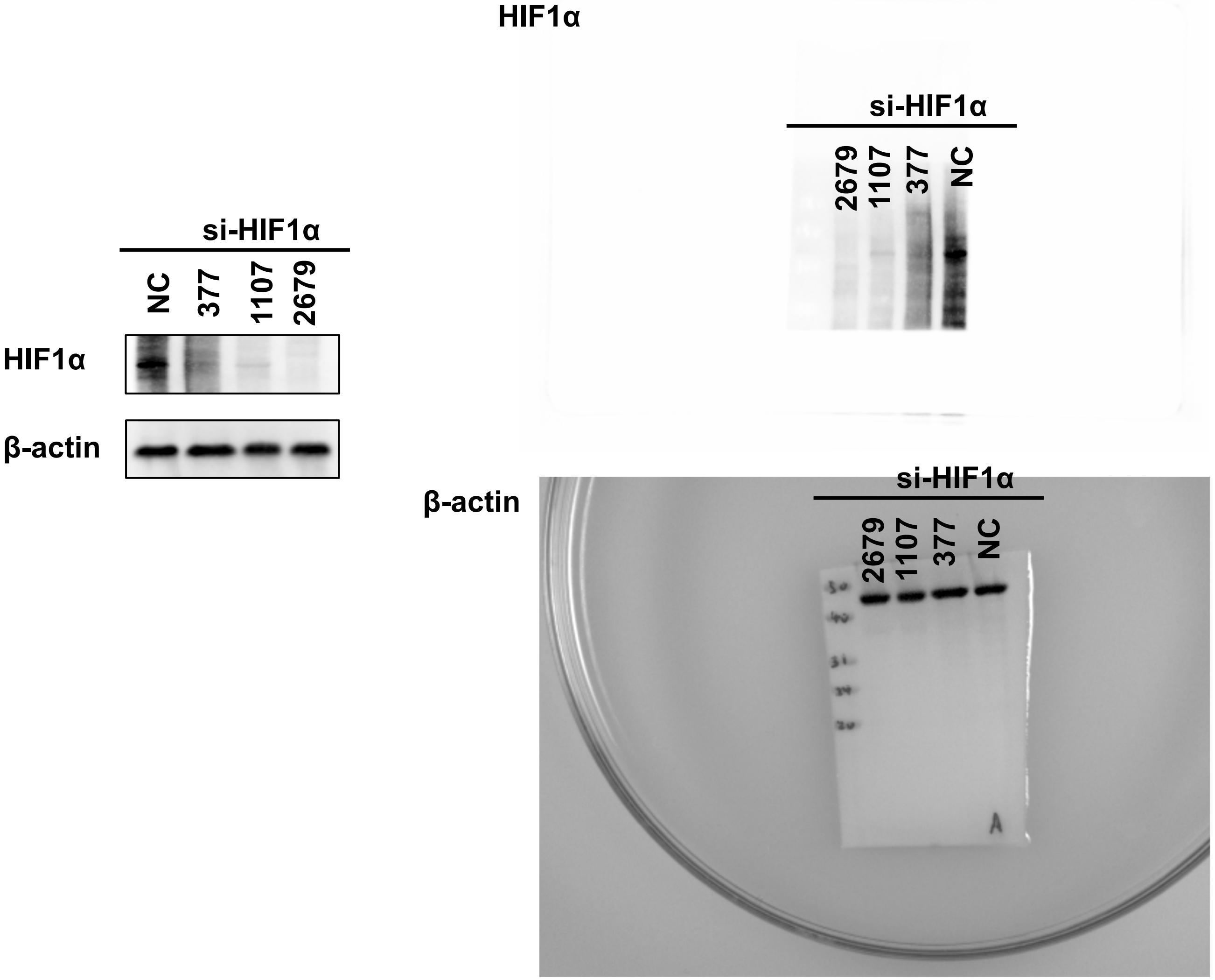

Supplementary Fig. 6C

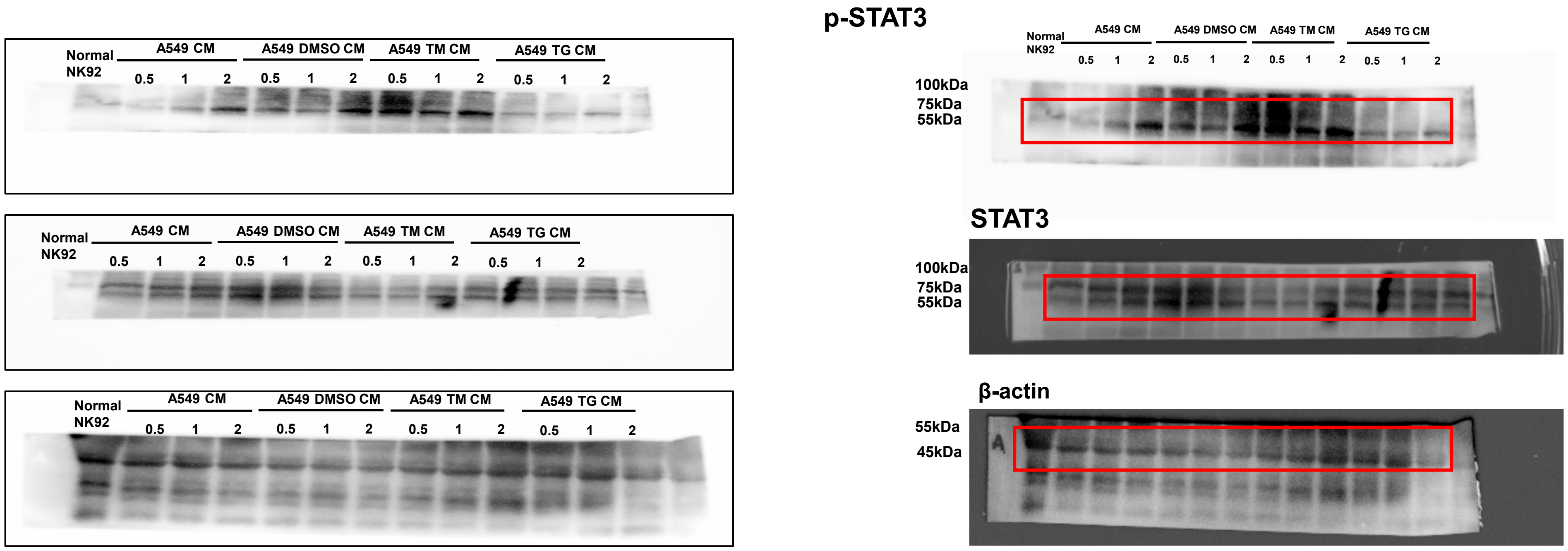

Fig. 5E

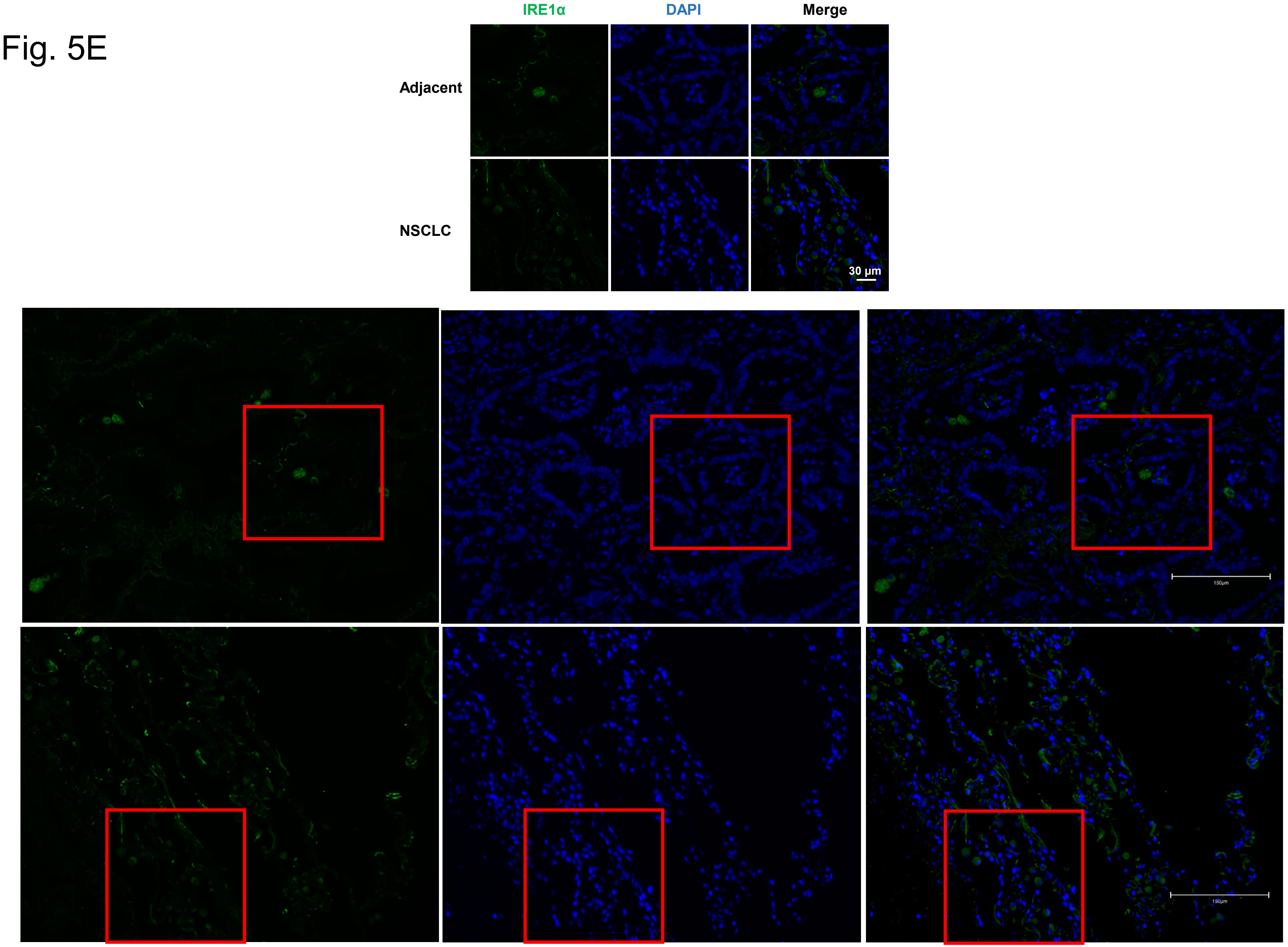

Fig. 5E

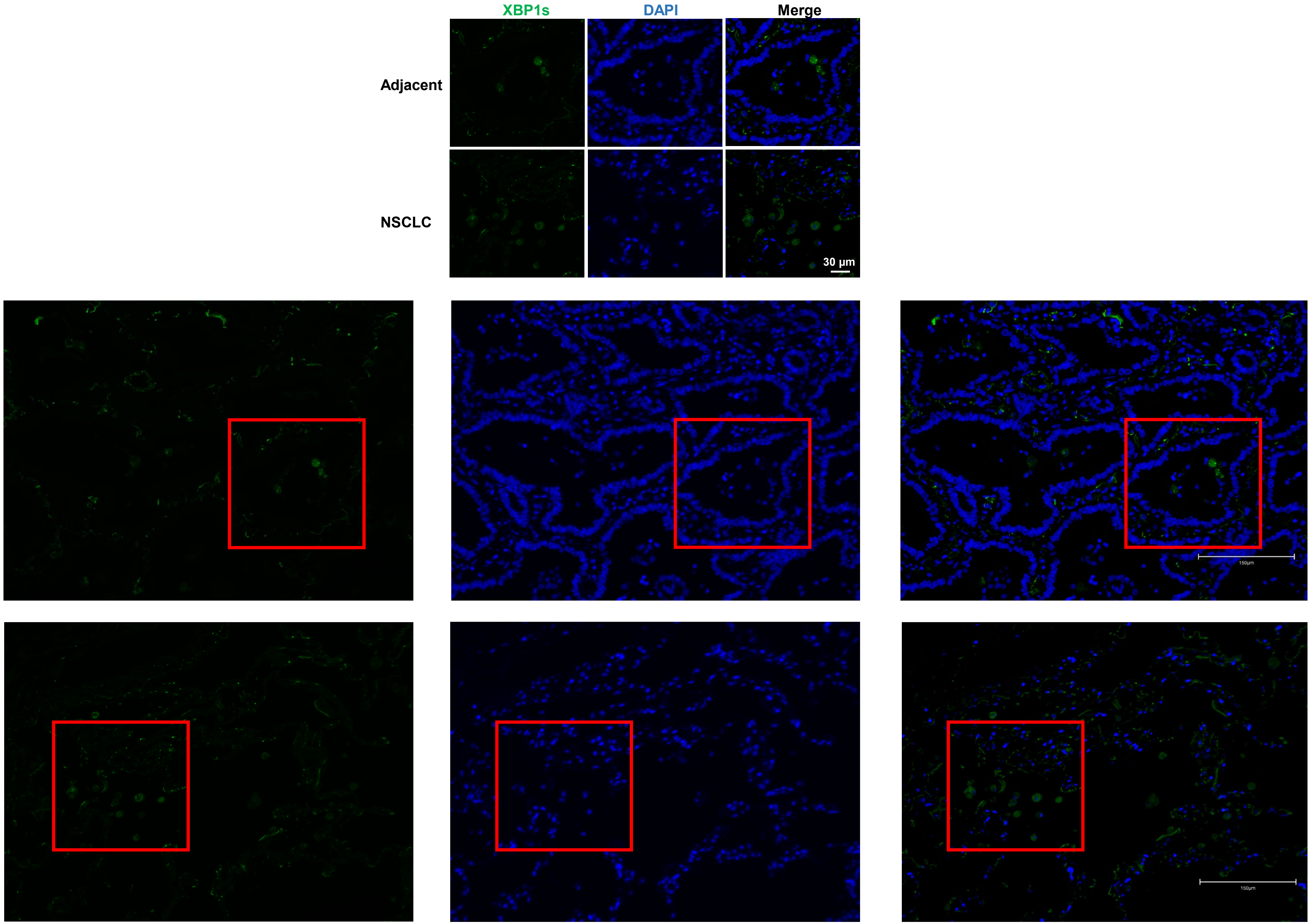

Fig. 7D

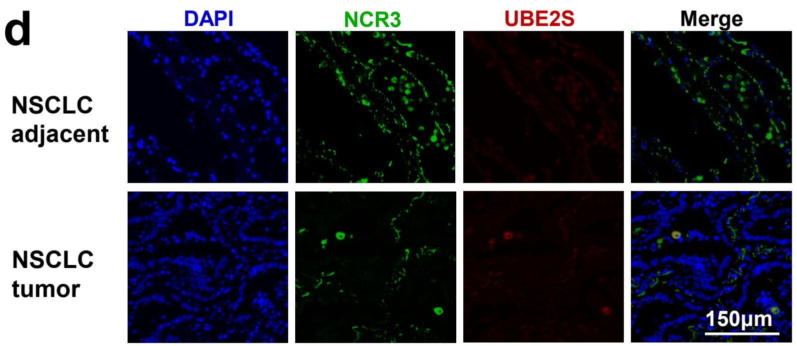

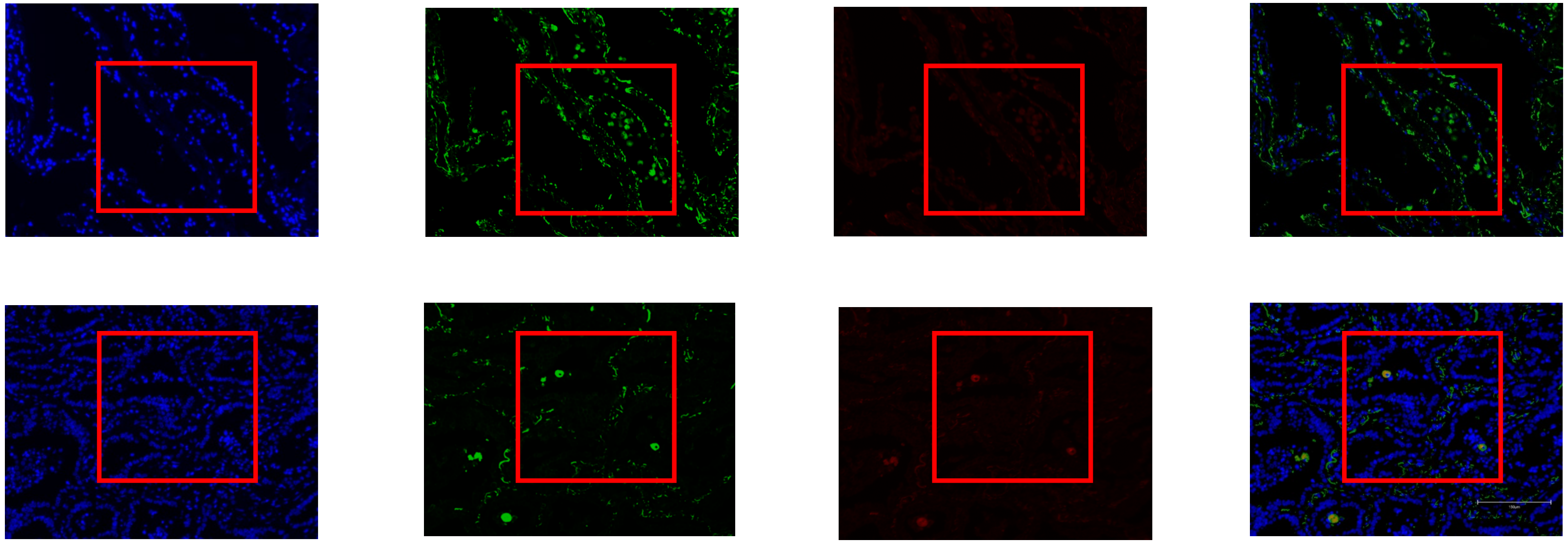

Figure S5

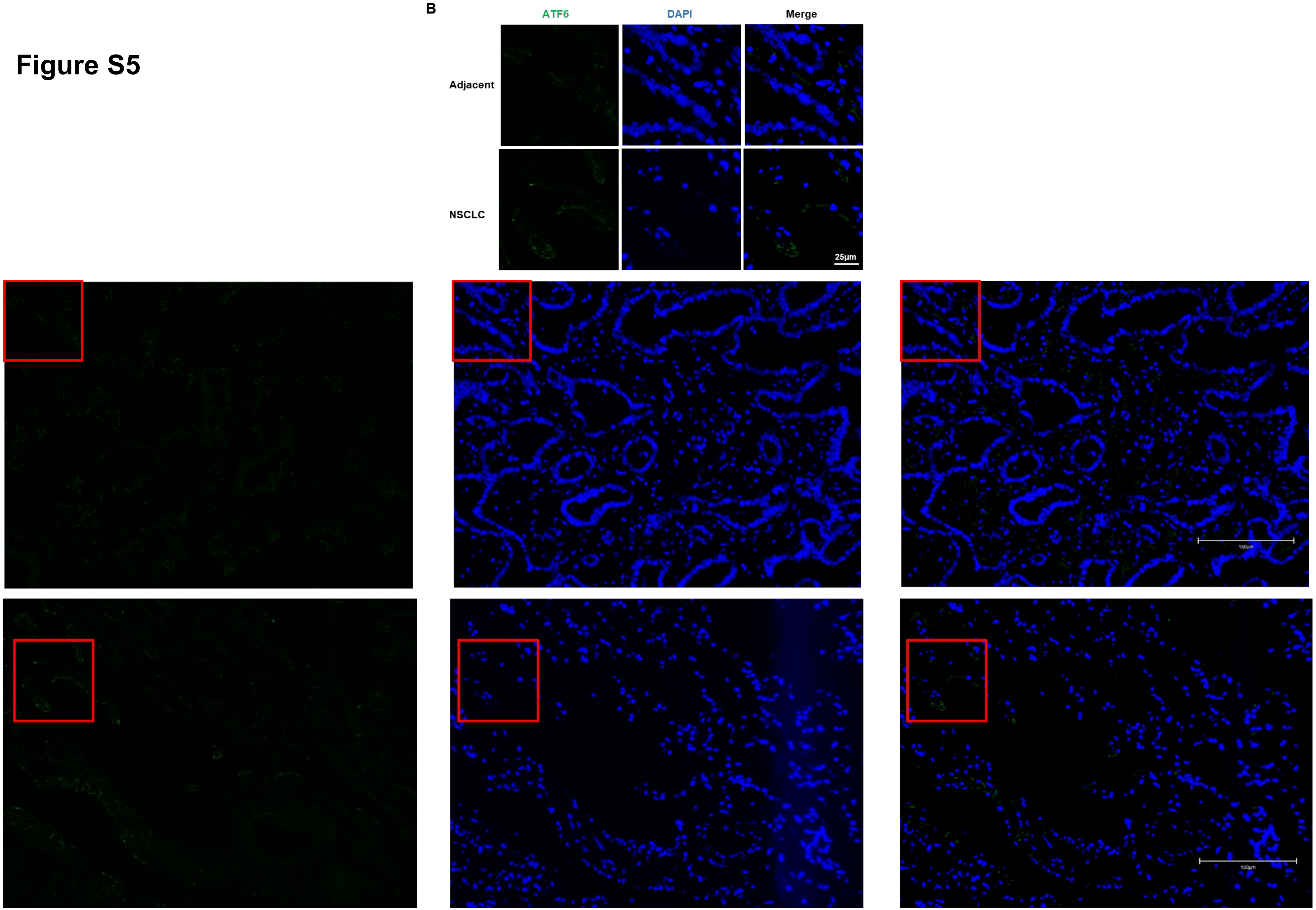

Figure S5

C

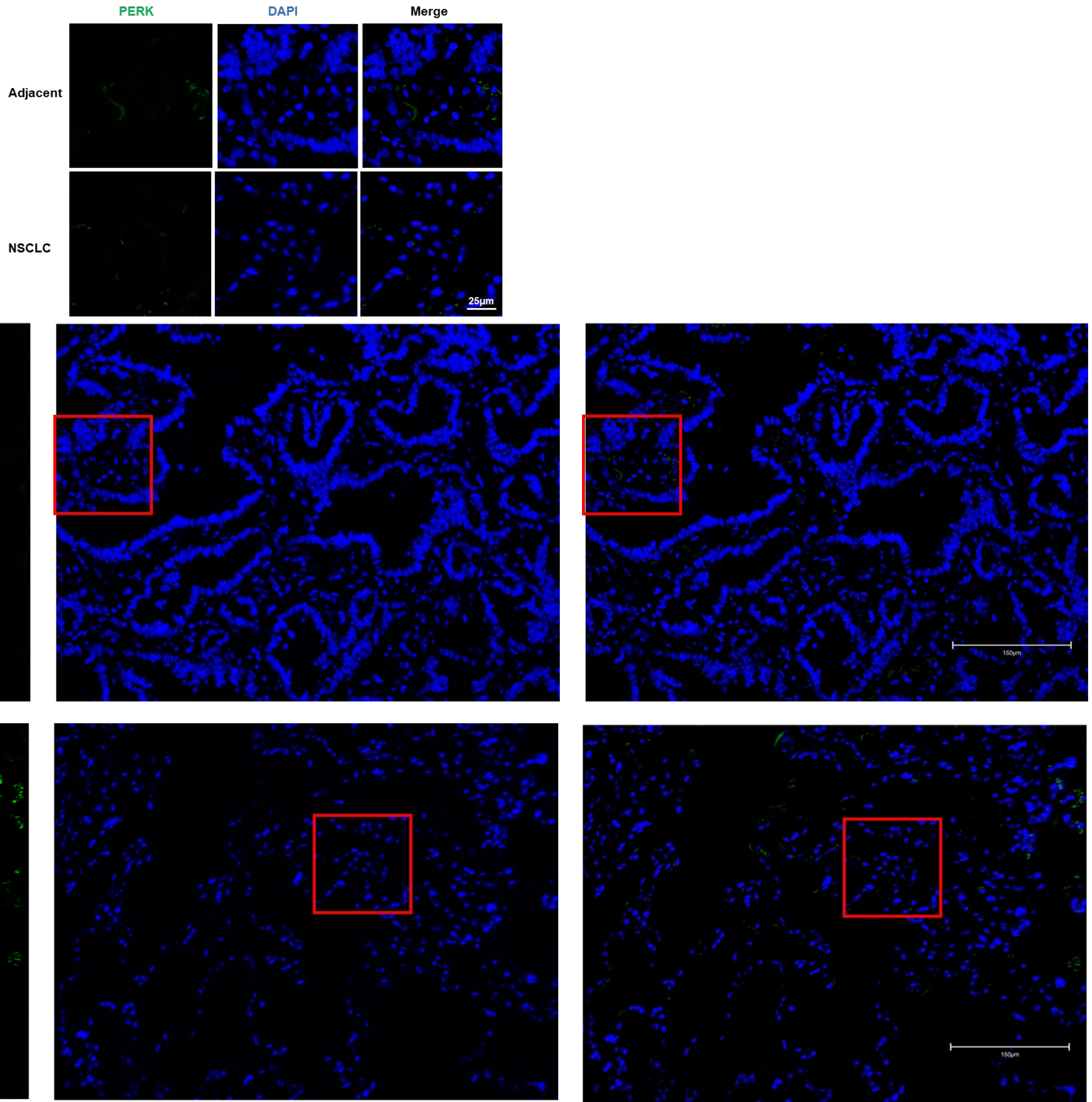

Supplement: Supplementary file 2 — Supplementary Information [file 41698_2025_1140_MOESM2_ESM.pdf]
